# Composite Mapping for Peptide‐Based Data Storage with Higher Coding Density and Fewer Synthesis Cycles

**DOI:** 10.1002/advs.202503790

**Published:** 2025-04-26

**Authors:** Anxun Zhang, Longjie Wang, Xiaowei Zhai, Yao Xiao, Yanchan Wu, Yongxi Zhao, Kai Liu, Ji‐Shen Zheng, Dong Chen

**Affiliations:** ^1^ Department of Medical Oncology The First Affiliated Hospital School of Medicine Zhejiang University Hangzhou Zhejiang 310003 P. R. China; ^2^ College of Energy Engineering and State Key Laboratory of Clean Energy Utilization Zhejiang University Hangzhou Zhejiang 310003 P. R. China; ^3^ Zhejiang Key Laboratory of Smart Biomaterials College of Chemical and Biological Engineering Zhejiang University Hangzhou Zhejiang 310027 P. R. China; ^4^ The First Affiliated Hospital of USTC MOE Key Laboratory for Membraneless Organelles and Cellular Dynamics Division of Life Sciences and Medicine University of Science and Technology of China Hefei Anhui 230001 P. R. China; ^5^ School of Electrical and Information Engineering Quzhou University Quzhou Zhejiang 324000 P. R. China; ^6^ Institute of Analytical Chemistry and Instrument for Life Science The Key Laboratory of Biomedical Information Engineering of Ministry of Education School of Life Science and Technology Xi'an Jiaotong University Xi'an Shaanxi 710049 P. R. China; ^7^ Department of Chemistry Tsinghua University Beijing 100084 P. R. China; ^8^ State Key Laboratory of Rare Earth Resource Utilization Changchun Institute of Applied Chemistry Chinese Academy of Sciences Changchun Jilin 130022 P. R. China

**Keywords:** composite mapping, data storage, mass spectrometry sequencing, solid‐phase peptide synthesis, statistical analysis

## Abstract

Peptides are natural information‐bearing mediums and are promising for high‐density data storage. However, conventional mapping of one amino acid (AA) to one binary code has limited the improvement of coding density by increasing the total number of different AAs. Here, a novel composite mapping strategy is developed, where each position in the peptide sequence is a composite letter consisting of several different AAs, and thousands of composite letters are available for mapping, thus breaking the limit of conventional mapping. When 20 different AAs are used, the coding density of six‐AAs composite mapping achieves 15 bits/letter, while conventional mapping only reaches 4 bits/AA. The whole process of encoding data into composite letter sequences, synthesizing composite letter sequences via solid‐phase peptide synthesis, sequencing composite letter sequences by mass spectrometry, and decoding data from composite letter sequences is successfully demonstrated for the first time. Composite mapping also demonstrates several distinct advantages, including high coding density, few synthesis cycles, high reliability against errors, low probability of homopolymers, and good compatibility with other encoding algorithms. The developed composite mapping strategy provides a novel way for peptide‐based data storage to increase the coding density and reduce the synthesis cycles, showing great potential for large‐scale data storage.

## Graphical abstract

A novel composite mapping strategy for peptide‐based data storage is presented, which enhances coding density by using composite letters made up of multiple amino acids. This approach significantly increases data encoding capacity, achieving 15 bits per letter. The method demonstrates high reliability, low error rates, and compatibility with other encoding systems, showing great potential for large‐scale data storage.

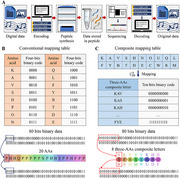

## 1 Introduction

The fast growth of information and the finite capacity of traditional storage mediums have triggered the pursuit of novel high‐density storage mediums. Natural information‐bearing mediums, such as DNA and peptides, are promising candidates to address the demand. DNA sequences carry natural genetic information. When used for data storage, DNA shows high storage density, long storage time, and low maintenance cost, and recent advancements in the synthesis and sequencing techniques of DNA have facilitated the development of DNA‐based data storage.^[^ ^1^ ^]^ The whole storage process, including encoding binary data into DNA sequences, synthesizing DNA sequences by solid‐phase synthesis, sequencing DNA sequences by second generation sequencing, and decoding DNA sequences into binary data, has successfully been demonstrated.^[^ ^2^ ^]^ However, because DNA‐based data storage relies solely on the 4 different nucleotides, A, T, C, and G, conventional mapping of one nucleotide to one binary code results in a maximum coding density of only 2 bits/nucleotide, which is inadequate for large‐scale data storage.^[^ ^3^ ^]^

Similar to DNA, peptides are also a natural information‐bearing medium and have a high storage density and a long lifespan.^[^ ^4^ ^]^ Compared with DNA, peptides have a larger number of amino acids (AAs), i.e., more than 20 different AAs.^[^ ^5^ ^]^ Therefore, three nucleotides determine one AA in nature, and the maximum coding density of peptide‐based data storage could increase to 5 bits/AA under conventional one‐to‐one mapping when 32 different AAs are used. The whole process of peptide‐based data storage, including encoding binary data into peptide sequences, synthesizing peptide sequences by solid‐phase synthesis, and sequencing peptide sequences by tandem mass spectrometry, has also been proven to be feasible.^[^ ^6^ ^]^ However, the advances in peptide‐based data storage are still limited due to the conventional one‐to‐one mapping strategy.^[^ ^7^ ^]^

Under the conventional one‐to‐one mapping strategy, the maximum coding density is $p=\left\lfloor \log_{2}\left(n\right)\;\right\rfloor$, where n is the total number of different AAs, and ⌊ ⌋ means rounding down. Therefore, the coding density is low, and increasing the coding density by increasing the total number of different AAs is very limited. In addition to the low coding density, conventional mapping also causes a high probability of homopolymers with the same AA in three consecutive positions, which will decrease the synthesis and sequencing accuracy.^[^ ^8^ ^]^ To correct possible errors, Reed‐Solomon (RS) and RaptorQ codes are often integrated in the encoding and decoding algorithms,^[^ ^2^, ^9^ ^]^ and redundancy is introduced at the same time, thus further reducing the coding density. To increase the coding density, various strategies, such as the Huffman algorithm, are developed. Recently, a strategy by exploiting the redundancy in synthesis and sequencing appears to be a breakthrough in improving the coding density.^[^ ^2e^ ^]^ Since multiple DNA sequences are synthesized at each time by solid‐state synthesis and multiple DNA sequences are sequenced at each time by second generation sequencing,^[^ ^10^ ^]^ probability information of 4 different nucleotides at each position could be obtained and the coding density increases 8 bits/letter by using the combination of 4 different nucleotides with predetermined probabilities to represent an 8‐bits binary code. Despite the advances, the developments of novel mapping strategies are highly desired to further increase the coding density and reduce the synthesis cycles for large‐scale data storage.

In this study, a novel composite mapping strategy is developed to substantially increase the coding density of peptide‐based data storage, thus reducing the synthesis cycles. Different from conventional one‐to‐one mapping, where one AA is mapped to one binary code, a composite letter consisting of several different AAs is mapped to one binary code. When 20 different AAs are used, the maximum coding density of conventional mapping is only 4 bits/AA, while the maximum coding density of six‐AAs composite mapping is 15 bits/letter, as the number of letters available for mapping increases from 20 to 38 760. Therefore, 176 bits of text data could be encoded into a single sequence of 12 six‐AAs composite letters under composite mapping. The whole process of storing and retrieving data into and from composite letters is successfully demonstrated. Each composite letter, i.e., a mixture of k different AAs for k‐AAs composite mapping, in the sequence is synthesized by solid‐phase peptide synthesis at one time. Each sequence of k‐AAs composite letters including multiple peptide chains is sequenced by mass spectrometry at one time, and statistical analysis is then performed to retrieve the sequence of composite letters. The composite mapping strategy also has a low probability of homopolymers and is compatible with other encoding algorithms, making it well suited for large‐scale data storage.

## 2 Results

### 2.1 Composite Mapping for Peptide‐Based Data Storage with Higher Coding Density and Fewer Synthesis Cycles

**Figure 1:**
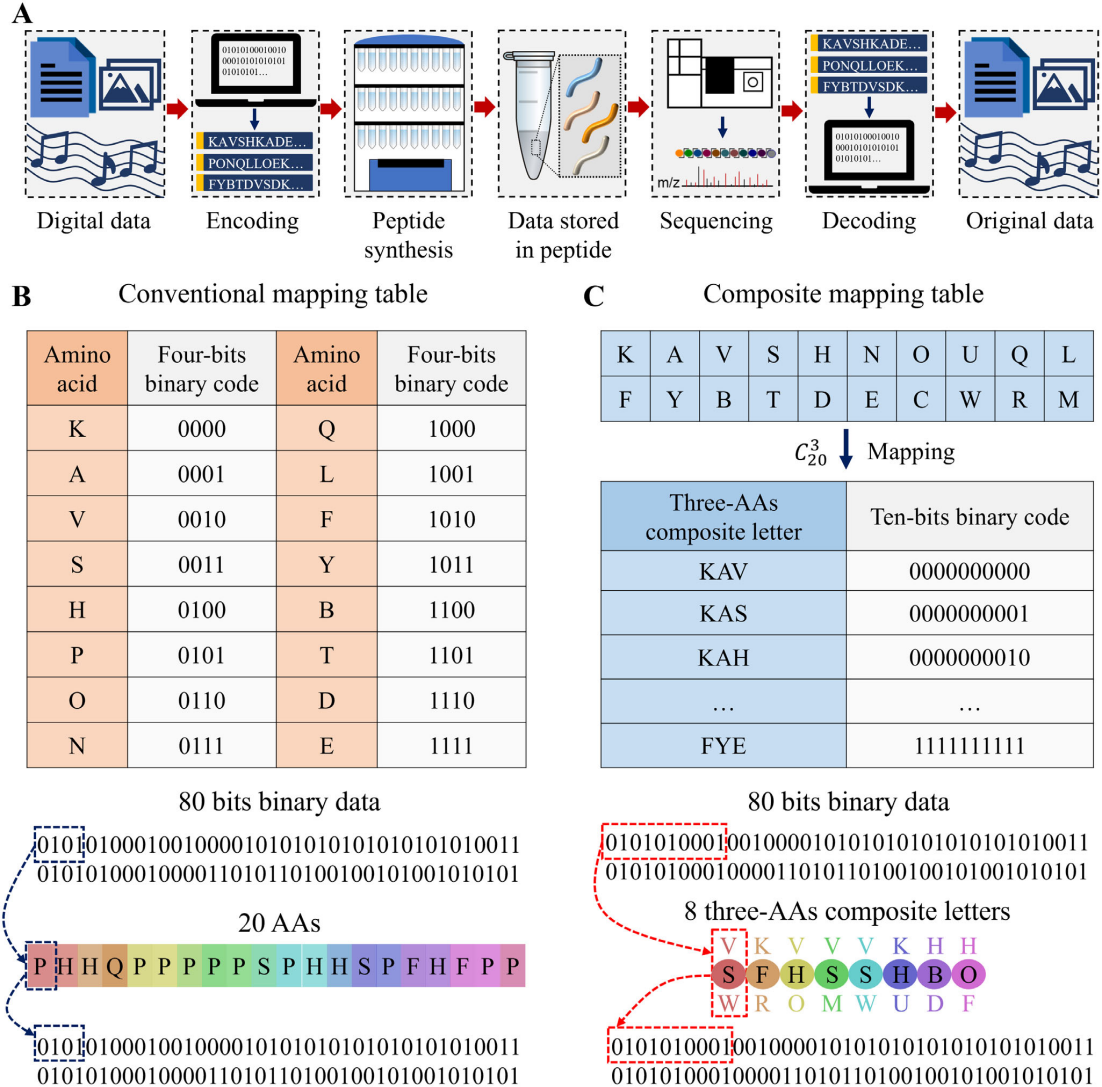
Data storage using composite mapping for higher coding density and fewer synthesis cycles. A) Flowchart of peptide‐based data storage, including encoding, peptide synthesis, storage, peptide sequencing, and decoding. Binary data is encoded into/decoded from peptide sequences according to a mapping table. B) Conventional mapping table of 2^4^ AAs to 2^4^ four‐bits binary codes. The maximum coding density is $p=\left\lfloor \log_{2}\left(20\right)\right\rfloor =4$, and each AA represents a four‐bits binary code. C) Composite mapping table of 2^10^ three‐AAs composite letters to 2^10^ ten‐bits binary codes. The maximum coding density is $p=\left\lfloor \log_{2}\left(C_{20}^{3}\right)\right\rfloor =10$, and each three‐AAs composite letter represents a ten‐bits binary code. A three‐AAs composite letter is a representation of a position in the peptide sequence that consists of three different AAs in a predetermined combination. Compared with conventional mapping, composite mapping has a higher coding density.

The flowchart of peptide‐based data storage includes encoding binary data into peptide sequences, synthesizing peptide sequences, storage, sequencing peptide sequences, and decoding binary data from peptide sequences, as shown in **Figure** **1**A. During the whole storage process, encoding/decoding binary data into/from peptide sequences is essential and a mapping table is indispensable. For conventional one‐to‐one mapping, the maximum coding density is $p=\left\lfloor \log_{2}\left(n\right)\;\right\rfloor$, where n is the total number of different AAs and ⌊ ⌋ means rounding down. When the total number of different AAs is n = 20, the maximum coding density is $p=\left\lfloor \log_{2}\left(20\right)\;\right\rfloor =4$. Therefore, 2^4^ AAs are selected and mapped to 2^4^ four‐bits binary codes, and each AA represents a four‐bits binary code, as shown in Figure 1B. To break the limit of the total number of different AAs, a composite mapping strategy is developed, where a combination of k different AAs regardless of order is selected from the total number of n different AAs to represent a position in the peptide sequence, and thus the maximum coding density is $p=\left\lfloor \log_{2}\left(C_{n}^{k}\right)\;\right\rfloor$, where k is the number of different AAs used for each composite letter and there are $C_{n}^{k}=\frac{n!}{k!(n-k)!}$ different kinds of combinations. When the total number of different AAs is n = 20 and the number of different AAs used for each composite letter is k = 3, the maximum coding density is $p=\left\lfloor \log_{2}\left(C_{20}^{3}\right)\;\right\rfloor =10$, which is much higher than that of conventional mapping. Therefore, 2^10^ three‐AAs composite letters are selected and mapped to 2^10^ ten‐bits binary codes, and each three‐AAs composite letter, which consists of three different AAs in a predetermined combination, represents a ten‐bits binary code, as shown in Figure 1C. For comparison, 80 bits binary data is mapped to 20 AAs under conventional mapping, while only 8 three‐AAs composite letters are required under composite mapping, which greatly increases the coding density and reduces the synthesis cycles.

### 2.2 Three‐AAs Composite Mapping

**Figure 2:**
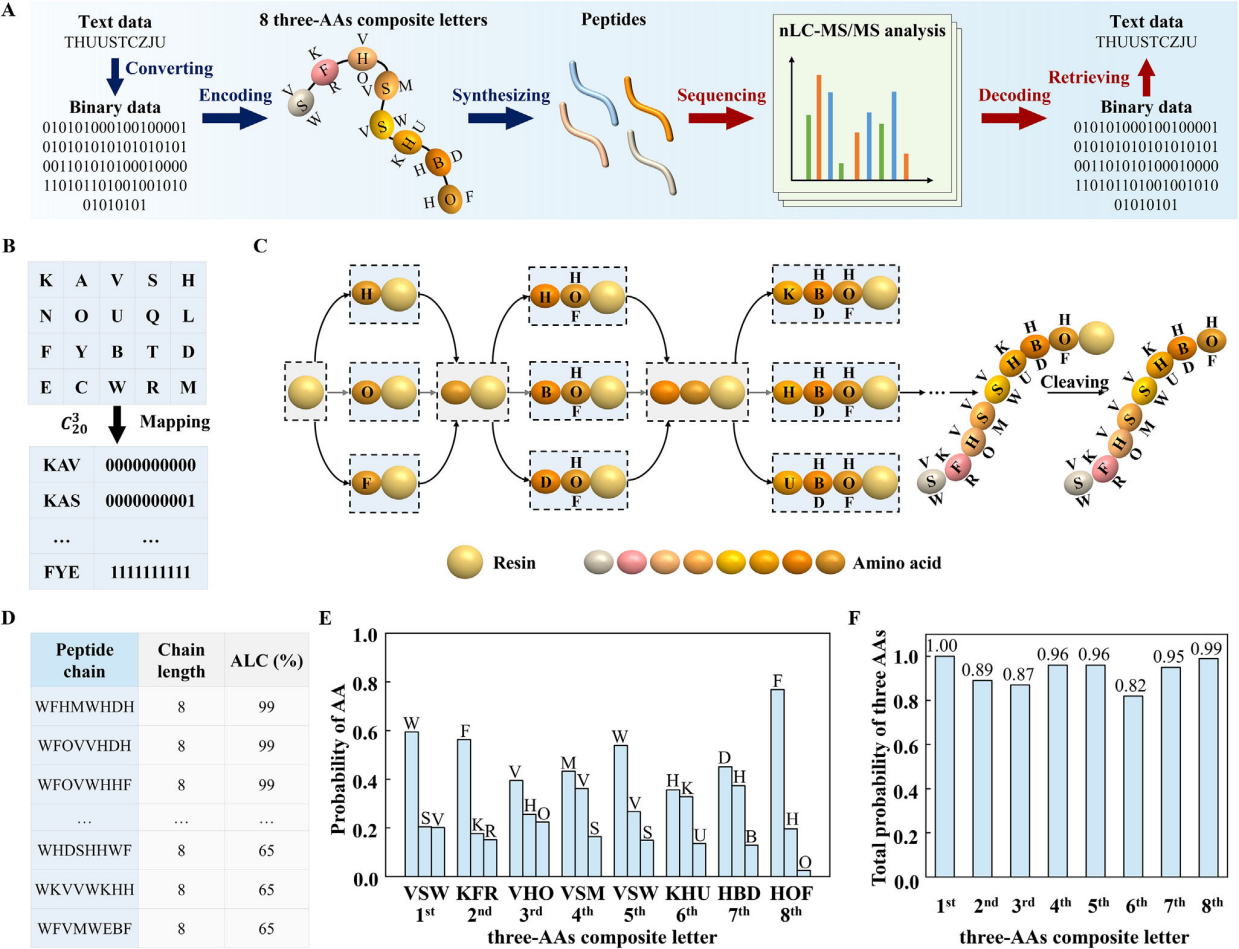
Storing and retrieving text data based on three‐AAs composite letters. A) Storing and retrieving text data “THUUSTCZJU” into and from a sequence of 8 three‐AAs composite letters, i.e., VSW, KFR, VHO, VSM, VSW, KHU, HBD and HOF. B) Mapping table between three‐AAs composite letters and ten‐bits binary codes. If not specified, 20 different AAs are used for composite mapping. C) Schematic diagram showing the strategy to synthesize the sequence of 8 three‐AAs composite letters using Fmoc solid‐phase peptide synthesis. The composite mapping strategy results in 3^8^ different peptide chains. D) Peptide chains obtained by mass spectrometry and screened by chain length = 8 and average local confidence (ALC)≥65%. 6442 peptide chains are left for statistical analysis. E) Probability of the three AAs with the top three highest probability at each position of the peptide sequence. The three‐AAs composite letter at each position is the combination of the three AAs with the top three highest probability at each position. A sequence of 8 three‐AAs composite letters, i.e., VSW, KFR, VHO, VSM, VSW, KHU, HBD, and HOF, is thus retrieved. F) Total probability of the three AAs in each composite letter.

To validate the feasibility of composite mapping, the whole process of storing and retrieving 80 bits of text data of “THUUSTCZJU” into and from a sequence of 8 three‐AAs composite letters is demonstrated, including converting, encoding, synthesizing, sequencing, decoding, and retrieving, as schematically shown in **Figure** **2**A. To construct the composite mapping table, 20 different AAs, including K, A, V, S, H, N, O, U, Q, L, F, Y, B, T, D, E, C, W, R and M, are selected by considering the factors of mass, solubility, cost, synthesis, and sequencing, as shown in Figures S1 and S2 (Supporting Information). For three‐AAs composite letters, the maximum coding density is $p=\left\lfloor \log_{2}\left(C_{20}^{3}\right)\;\right\rfloor =10$, and thus 2^10^ three‐AAs composite letters are selected from the $C_{20}^{3}$ combinations and mapped to 2^10^ ten‐bits binary codes, shown in Figure 2B and Figure S3 (Supporting Information). In the composite mapping table, three different AAs are selected consecutively from left to right of K, A, V, S, H, N, O, U, Q, L, F, Y, B, T, D, E, C, W, R and M. As the three‐AAs composite letter changes from KAV to KAS, KAH…FYT, FYD, and FYE, the ten‐bits binary code increases from 0 000 000 000 to 0 000 000 001, 0 000 000 010…1 111 111 101, 1 111 111 110 and 1 111 111 111. The 80 bits text data are then encoded into a sequence of 8 three‐AAs composite letters according to the composite mapping table, i.e., VSW, KFR, VHO, VSM, VSW, KHU, HBD, and HOF.

The sequence of 8 three‐AAs composite letters is synthesized by Fmoc solid‐phase peptide synthesis (SPPS).^[^ ^11^ ^]^ For each composite letter, three different AAs with the same mole mass are mixed and coupled to the ends of peptide chains at the same time by removing the Fmoc group, as shown in Figures 2C and Figures S4–S7 (Supporting Information). Alternatively, three different AAs with the same mole mass are coupled separately to the ends of peptide chains by split‐pool Fmoc SPPS in three different tubes, as shown in Figure S8 (Supporting Information). Due to the difference in structure, the coupling efficiencies of different AAs to different ends are different, especially when they are coupled to the ends at the same time. However, the difference won't affect the retrieval of correct composite letters, as the retrieval of three‐AAs composite letters relies on the statical analysis of AAs with the top three highest probability at each position, which is proved to be feasible by the following experiments.

Unlike conventional mapping, where each position in the peptide sequence corresponds to a specific AA, composite mapping results in three different AAs at each position. For example, the AA at the 1^st^ position could be V, S, or W, and the AA at the 2nd position could be K, F, or R. Theoretically, there are up to 3^8^ different chains in the synthesized peptide sample. Mass spectra of different AA chains are obtained by nano‐liquid chromatography/tandem mass spectrometry (nLC‐MS/MS), as shown in Figure S9 (Supporting Information), and thousands of peptide chains with detailed information, such as chain length, average local confidence (ALC), mass/charge ratio and so on, are analyzed by the software PEAKS X+, as shown in Figures S10 and S11 (Supporting Information). Each three‐AAs composite letter is retrieved from the combination of the three AAs with the top three highest probability at each position. Due to possible noises and errors in synthesis and sequencing, ALC is an important indicator to assess the reliability of obtained peptide chains, and higher ALC indicates higher reliability. However, when ALC is too high, i.e., ALC≥85%, errors occur in the three‐AAs composite letters containing U (diaminobutanoic acid), as shown in Figure S12 (Supporting Information). This is because U is a non‐canonical AA, which exhibits poor sequencing accuracy, and too many peptide chains are screened out with only 2787 chains left for statistical analysis when ALC≥85%. When ALC≥75% or lower, no errors are observed, as more than 4452 chains are statistically analyzed. Interestingly, the 8 three‐AAs composite letters retrieved are all correct even when ALC≥5%, suggesting that the results obtained by statistical analysis are robust against noises and errors.

After screening the peptide chains by chain length = 8 and ALC≥65%, 6442 peptide chains are left for statistical analysis, as shown in Figure 2D. The three‐AAs composite letter at each position is retrieved from the three AAs with the top three highest probability at each position, and the sequence of 8 three‐AAs composite letters, i.e., VSW, KFR, VHO, VSM, VSW, KHU, HBD and HOF, is thus obtained, as shown in Figure 2E. Though equal amounts of AAs are used in the synthesis process, the three AAs retrieved at each position generally don't have an equal probability. This is because the efficiencies of coupling different AAs to different ends are different. Nevertheless, the three AAs with the top three highest probability at each position are robust against the complexity and the total probability of the three AAs at each position is close to 1, as shown in Figure 2F. The 80 bits of text data are then obtained by decoding each three‐AAs composite letter back to a ten‐bits binary code according to the composite mapping table.

The synthesis and sequencing of each composite letter take advantage of the fact that multiple peptide chains are synthesized at each time and multiple peptide chains are sequenced at each time. Since the retrieval of composite letters relies on the statistical analysis, i.e., the probability of AAs at each position, it doesn't require that every possible chain is synthesized and every possible chain is sequenced. For example, the sequence of 8 three‐AAs composite letters theoretically contains 3^8^ = 6561 different peptide chains. However, a minimum of only 4452 chains are required to retrieve the correct sequence. In addition, the statistical analysis also makes the results reliable and robust to errors.

### 2.3 Six‐AAs Composite Mapping

**Figure 3:**
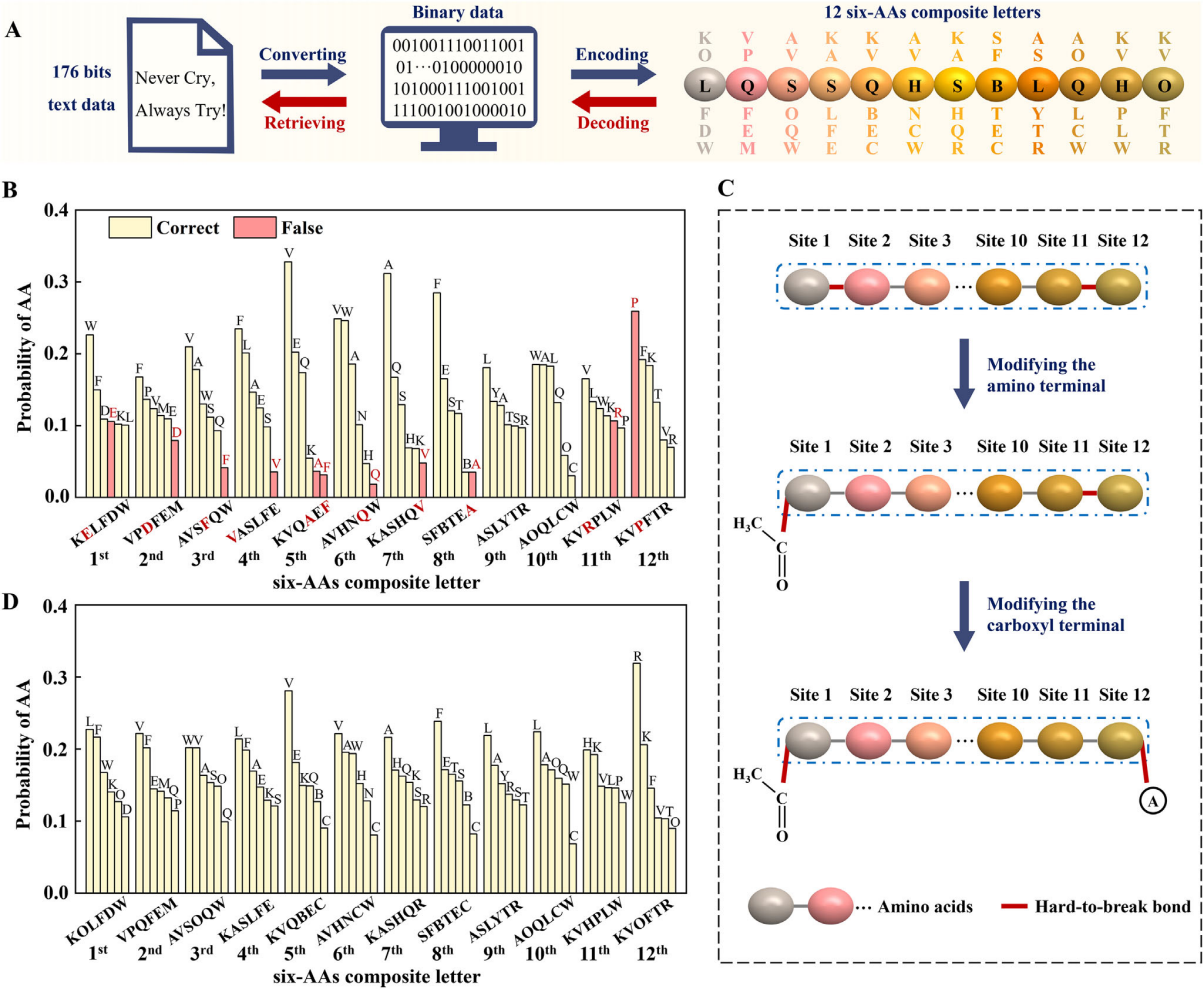
Storing and retrieving text data based on six‐AAs composite letters. A) Encoding and decoding binary data “Never Cry, Always Try!” into and from a sequence of 12 six‐AAs composite letters, i.e., KOLFDW, VPQFEM, AVSOQW, KASLFE, KVQBEC, AVHNCW, KASHQR, SFBTEC, ASLYTR, AOQLCW, KVHPLW and KVOFTR. Each six‐AAs composite letter represents a fifteen‐bits binary code. B) Six‐AAs composite letters retrieved from the six AAs with the top six highest probability at each position before terminal modifications. Errors appear in the retrieved six‐AAs composite letters due to the hard‐to‐break bonds at the two terminals. C) Schematic diagram showing the modifications of the amino terminal with an acetyl group and the carboxyl terminal with an alanine. D) Six‐AAs composite letters retrieved at each position after terminal modifications. The modifications of the two terminals improve the accuracy of sequencing by mass spectrometry. 10 590 peptide chains are left for statistical analysis after screening by chain length = 12 and ALC≥65%.

Compared with three‐AAs composite mapping, the maximum coding density of six‐AAs composite mapping with the same total number of 20 different AAs is even higher, and $p=\left\lfloor \log_{2}\left(C_{20}^{6}\right)\;\right\rfloor =15$. 176 bits text data of “Never Cry, Always Try!” could be encoded/decoded into/from a single sequence of 12 six‐AAs composite letters, as shown in **Figure** **3**A. In the six‐AAs composite mapping table, 2^15^ six‐AAs composite letters are selected from the $C_{20}^{6}$ combinations and mapped to 2^15^ fifteen‐bits binary codes, as shown in Figure S13 (Supporting Information). The synthesis and sequencing of the 12 six‐AAs composite letters follow the same procedure as the 8 three‐AAs composite letters. Peptide chains obtained by mass spectrometry are screened by chain length = 12 and ALC≥65%, as shown in Figures S14–S16 (Supporting Information). However, errors appear in the six‐AAs composite letters retrieved from the six AAs with the top six highest probability at each position, as shown in Figure 3B. This is because longer chain lengths will increase the complexity of peptide synthesis and sequencing. Especially, the bonds at the two terminals of the peptide chain are hard to break during tandem mass spectrometry,^[^ ^12^ ^]^ and the problem becomes worse when the chain length becomes longer.^[^ ^13^ ^]^ To solve the problem, the amino terminal at the 1st position and the carboxyl terminal at the 12th position are modified with an acetyl group and an alanine, respectively, as shown in Figure 3C. After modifying the amino terminal with an acetyl group, the bond at the 1st position becomes easy to break and only an error appears near the carboxyl terminal at the 12th position, as shown in Figure S17 (Supporting Information). When both terminals are modified, the bonds in the 1st and 12th positions become easy to break during tandem mass spectrometry, as they are no longer terminal bonds. The subsequent statistical analysis yields accurate results, and the 12 six‐AAs composite letters, which are retrieved by following the same procedure as the 8 three‐AAs composite letters (Figures S18–S20, Supporting Information), are all correct, as shown in Figure 3D. In addition, the results are reliable and robust, when ALC≥95%, ALC≥75%, ALC≥55%, or ALC≥35%, as shown in Figure S21 (Supporting Information).

**Figure 4:**
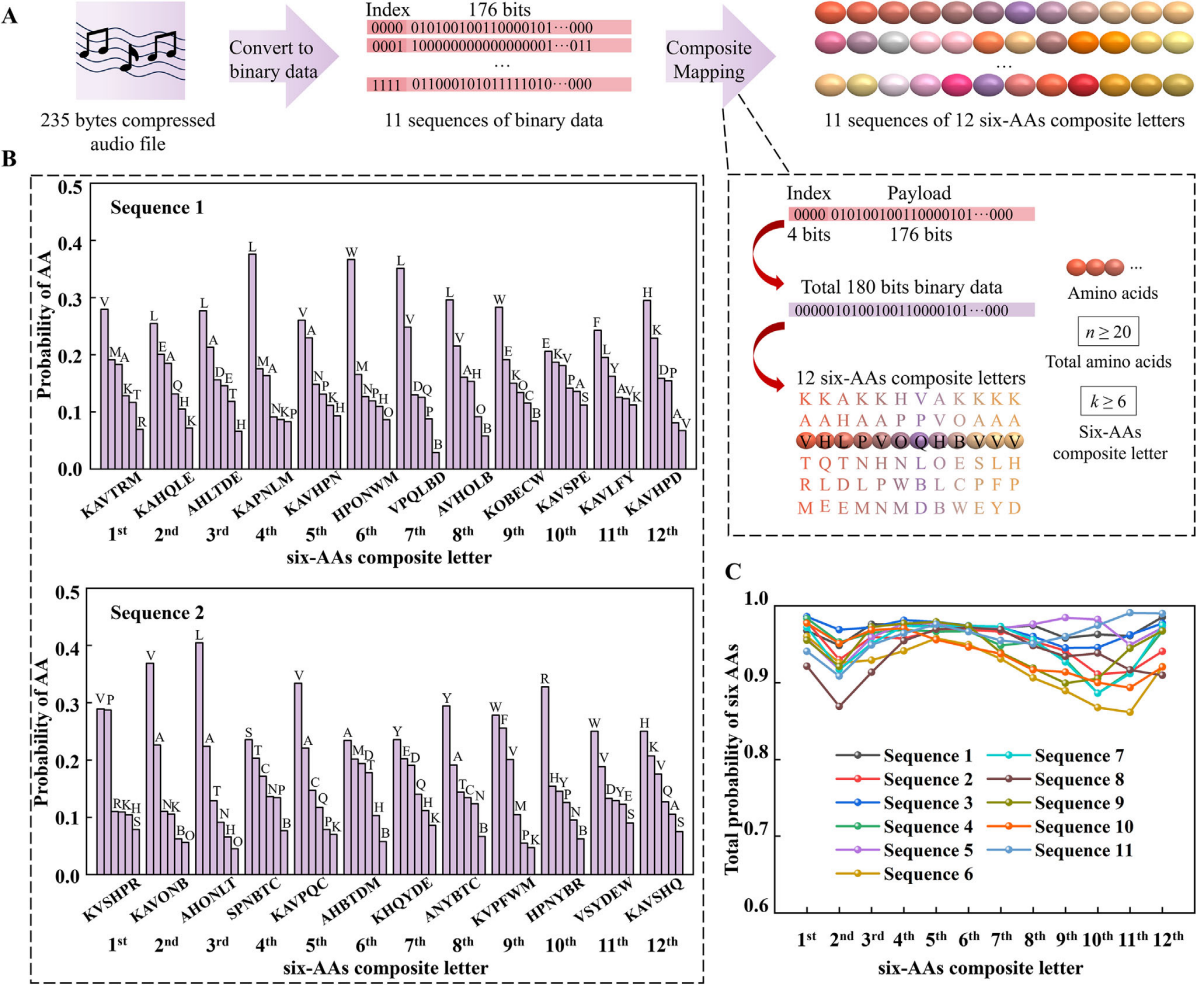
Storing and retrieving audio data based on six‐AAs composite letters. A) 235 bytes of compressed audio data are divided into 11 sequences of 176 bits binary data with 4 bits index, which are encoded into 11 sequences of 12 six‐AAs composite letters. B) The 1st and 2nd sequences were retrieved by statistical analysis. No errors are observed in all the 11 sequences of 12 six‐AAs composite letters. C) Total probability of the six AAs in each composite letter of the 11 sequences.

The developed composite mapping strategy is superior in increasing the coding density and reducing the synthesis cycles, making it advantageous for large‐scale data storage. 235 bytes of compressed audio data could be divided into 11 sequences of 176 bits binary data with 4 bits index and then encoded into only 11 sequences of 12 six‐AAs composite letters, as shown in **Figure** **4**A. By following the same procedure as the 12 six‐AAs composite letters for text data, 11 sequences of 12 six‐AAs composite letters are successfully synthesized and the 12 six‐AAs composite letters in each sequence are retrieved correctly, as shown in Figure 4B and Figure S22 (Supporting Information). The total probability of the six AAs in each composite letter is larger than 85%, suggesting good reliability of the composite mapping strategy, as shown in Figure 4C.

### 2.4 Performances of Composite Mapping

**Figure 5:**
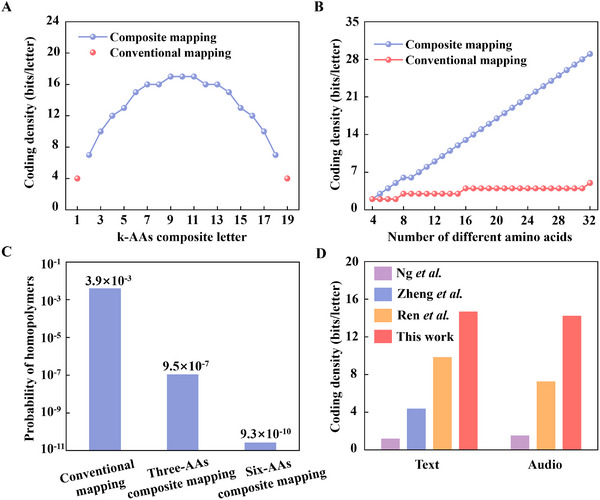
Performances of composite mapping. A) Coding density using k‐AAs composite letters. The total number of different AAs is 20. The coding density using conventional mapping is only 4 bits/letter. B) Maximum coding density using conventional mapping (red dots) and n/2‐AAs composite mapping (blue dots) with a total number of n different AAs. C) Probability of homopolymers with the same letter in three consecutive positions using conventional mapping, three‐AAs composite mapping, and six‐AAs composite mapping. The total number of different AAs is 20. D) Comparison of the coding density of peptide‐based data storage between previous works and this work.^[^ ^7^, ^9^ ^]^

Composite mapping significantly broadens the pool of composite letters available for mapping, thus enhancing the coding density and reducing the synthesis cycles. For a total number of 20 different AAs, the maximum coding density $p=\left\lfloor \log_{2}\left(C_{20}^{k}\right)\;\right\rfloor$ strongly depends on the number of different AAs used for each composite letter and reaches a maximum value of $p=\left\lfloor \log_{2}\left(C_{20}^{10}\right)\;\right\rfloor =17$, while the maximum coding density of conventional mapping is limited to $p=\left\lfloor \log_{2}\left(20\right)\;\right\rfloor =4$, as shown in **Figure** **5**A. The difference between composite mapping and conventional mapping becomes larger, as the total number of different AAs increases, as shown in Figure 5B. When the total number of different AAs reaches 32, the maximum coding density of conventional mapping is only $p=\left\lfloor \log_{2}\left(32\right)\;\right\rfloor =5$, while the maximum coding density of sixteen‐AAs composite mapping is $p=\left\lfloor \log_{2}\left(C_{32}^{16}\right)\;\right\rfloor =29$.

In addition to the high coding density, composite mapping also significantly lowers the probability of homopolymers with the same letter in three consecutive positions, as shown in Figure 5C. The appearance of homopolymers often compromises the accuracy of synthesis and sequencing, and the probability of homopolymers strongly depends on the coding density as (1/2^p^)^2^. For conventional mapping, the probability of homopolymers is (1/2^4^)^2^, and complex algorithms are often required to avoid the appearance of homopolymers. In contrast, the appearance of homopolymers under six‐AAs composite mapping is only (1/2^15^)^2^, which is much lower. In addition, the higher coding density and thus shorter chain length under composite mapping will further reduce the probability of homopolymers.

Composite mapping shows a good performance in improving the coding density and lowering the synthesis cycles. The coding density of p = 15 by six‐AAs composite mapping outperforms other reported peptide‐based data storage, as shown in Figure 5D. In addition, the composite mapping strategy is compatible with other algorithms, such as the Huffman algorithm, which could further improve the coding density.

## 3 Discussion

Compared with conventional mapping of one AA to one binary code, composite mapping, where each position in the peptide sequence is a composite letter consisting of several different AAs, demonstrates several distinct advantages: 1) High coding density. The number of composite letters available for composite mapping is thousands of times that available for conventional mapping. 2) Few synthesis cycles. Synthesizing one composite letter takes the same process as synthesizing one AA, and thus the high coding density could greatly reduce the synthesis cycles. 3) High reliability against errors. The retrieval of composite letters relies on the statistical analysis of thousands of peptide chains and thus is robust against noises and errors. 4) Low probability of homopolymers. Because of the huge number of composite letters, the appearance of homopolymers in composite mapping is negligible. 5) Good compatibility with other encoding algorithms. As composite mapping only replaces conventional mapping, composite mapping is compatible with other encoding algorithms, such as the Huffman algorithm, to further increase the encoding density.

## 4 Conclusion

A novel composite mapping strategy is developed for peptide‐based data storage to greatly increase the coding density and reduce the synthesis cycles. While conventional mapping maps one AA to one binary code, composite mapping maps a composite letter consisting of several different AAs to one binary code, which increases the number of composite letters available for mapping thousands of times. The feasibility of composite mapping is confirmed through the whole process of peptide‐based data storage, including encoding data into composite letter sequences, synthesizing composite letter sequences via solid‐phase peptide synthesis, sequencing composite letter sequences by mass spectrometry, and decoding data from composite letter sequences. Storing and retrieving text and audio data into and from three‐AAs and six‐AAs composite letters are successfully demonstrated. Compared with conventional mapping, composite mapping demonstrates several distinct advantages, including high coding density, few synthesis cycles, high reliability against errors, low probability of homopolymers, and good compatibility with other encoding algorithms. The developed composite mapping strategy provides a novel solution to large‐scale data storage and will intrigue more innovations to further increase the coding density and reduce the synthesis cycles.

## Conflict of Interest

The authors declare no conflict of interest.

## Supporting information



Supporting Information

## Data Availability

The data that support the findings of this study are available in the supplementary material of this article.
