# Supplementary material for: Composite Mapping for Peptide‐Based Data Storage with Higher Coding Density and Fewer Synthesis Cycles
Source: Adv Sci (Weinh). 2025 Apr 26;12(27):2503790. doi: 10.1002/advs.202503790 (PMC12279161; doi:10.1002/advs.202503790)
Supplement: Supplementary file 1 — Supporting Information [file ADVS-12-2503790-s001.docx]

Supporting Information

Composite mapping for peptide-based data storage with higher coding density and fewer synthesis cycles

Anxun Zhang, Longjie Wang, Xiaowei Zhai, Yao Xiao, Yanchan Wu, Yongxi Zhao, Kai Liu, Ji-Shen Zheng*, Dong Chen*

**1. Selection of Amino Acids**

20 amino acids were selected for composite mapping by considering mass, solubility, cost, synthesis and sequencing. Since tandem mass spectrometry could not distinguish between leucine (L) and isoleucine (I), due to their identical molecular weights, only L was selected. Glycine (G) was omitted, as its small molecular weight caused frequent errors during sequencing by tandem mass spectrometry. Instead, non-canonical AAs, ornithine (O) and 2,3-diaminopropionic acid (B) were selected as replacements. Non-canonical AAs are advantageous due to their hydrophilicity, affordability and facilitation for synthesis and sequencing.

Researches indicated that during peptide fragmentation by tandem mass spectrometry, the amide bonds at the terminals of the peptide chain are particularly resistant to cleavage, which would lead to incorrect sequencing of terminal AAs, especially at the peptide's C-terminus or N-terminus. In addition, a fragment, such as PR, might be misread as RP.

De novo sequencing software PEAKS X+, which was optimized for canonical AAs, was used for statistical analysis. Extensive data on canonical AAs were analyzed and a series of fingerprints were created, which could improve the accuracy of de novo sequencing. In contrast, non-canonical AAs, which lacked sufficient data in the software, required post-translational modifications through canonical AAs.

Due to its unsatisfactory sequencing performance, non-canonical AA, diaminobutanoic acid (U), was preplaced by canonical AA, proline (P), for six-AAs composite mapping. Subsequent experiments demonstrated that P worked well for the synthesis and sequencing of six-AAs composite letter sequences.

**2. Materials and Methods**

**2.1. Materials**

Fmoc-Rink amide AM resin was purchased from Tianjin Nankai Hecheng (China). Fmoc-amino acids and O-(7-azabenzotriazol-1-yl)-N,N,N’,N’-tetramethyluronium hexafluorophosphate (HATU) were purchased from GL Biochem (China). Triisopropylsilane (TIPS), trifluoroacetic acid (TFA), 2-iodoacetamide (IAM), dl-1,4-dithiothreitol (DTT), formic acid (FA) and 1,2-ethanedithiol (EDT) were purchased from J&K Scientific (China). Thioanisole, N-methylmorpholine (NMM), acetic anhydride (Ac_2_O), piperidine and diethyl ether (Et_2_O) were purchased from Sinoreagent (China). N,N-diisopropylethylamine (DIEA) was purchased from Adamas (China). Water (LC-MS grade) and acetonitrile (LC-MS grade) were purchased from Merck (China). Dichloromethane (DCM) and N,N-dimethylformamide (DMF) were purchased from Chronchem (China).

The synthesis of peptides was performed using 0.1 mmol Fmoc-Rink amide AM resin (0.31 mmol/g Fmoc-Rink amide) via SPPS. First, the Fmoc-Rink amide AM resin was treated with 20% piperidine in DMF twice at 37°C for 5 minutes to remove the Fmoc protecting group. Then, the resin was suspended in DMF, and AA couplings were performed by adding 5 mL solution of 1 mmol Fmoc-amino acid, 0.98 mmol HATU and 2 mmol DIEA in DMF for 30 minutes at 37°C. Next, the resin coupled with AAs was washed thrice with DMF, thrice with DCM and thrice with DMF. After washing, the resin was treated with 20% piperidine in DMF twice at 37°C for 5 minutes to remove the Fmoc protecting group for the coupling of the next AA. These steps were repeated until the target sequence of composite letters was synthesized.

**2.2. N-terminal acetylation of peptides**

N-terminal acetylation of peptides was performed by adding the solution of 10% Ac_2_O and 10% DIEA in DMF. The reaction was maintained for 30 minutes at 37 °C and repeated once. Then the resin bearing the peptides was washed thrice with DMF, thrice with DCM and thrice with DMF.

**2.3. Peptide cleavage and global deprotection**

First, 0.01 mmol peptidyl resin bearing the peptides was treated with 5 mL solution of 2.5 vol% H_2_O, 2.5 vol% TIPS, 2.5 vol% EDT and 2.5 vol% thioanisole in TFA for 2 hours at room temperature for peptide cleavage and global deprotection. Second, the solution was concentrated by N_2_ blowing and then washed with cold diethyl ether. Third, the precipitates were centrifuged and washed with cold diethyl ether twice. Finally, the precipitates, which were the peptides encoded with data, were dried at room temperature.

**2.4. Sequencing of peptides**

The sequencing of peptides was divided into two steps, nano LC-MS/MS analysis and de novo peptide sequencing using PEAKS X+.

Before nano LC-MS/MS analysis, 100 μg peptides encoded with data were dissolved in 40 μL LC-MS-grade water. Then, 5 μL of 200 mM DTT in LC-MS-grade water was added to the peptide solution to react at room temperature for 10 minutes. Next, 5 μL of 200 mM IAM in LC-MS-grade water was added to the peptide solution, which was then incubated in dark at room temperature for 10 minutes. Finally, the peptide solution was centrifuged at 10,000 rpm for 5 minutes, and the supernatant was collected for subsequent nano LC-MS/MS analysis.

1 μL collected supernatant was used for nano LC-MS/MS analysis, which was performed on Thermo Fisher Q Exactive Plus Hybrid Quadrupole-Orbitrap Mass Spectrometer coupled to Thermo Fisher EASY-nLC 1200 System equipped with Acclaim PepMap RSLC C18 column (inner diameter=50 μm×length=15 cm, silica diameter=2 μm, and pore diameter=100 Å). The standard nano-LC method was run at 40 °С and a flow rate of 1 μL/min with the following gradient: 1% solvent B’ (solvent B’ is 0.1% FA and 20% DI water in acetonitrile) in solvent A’ (solvent A’ is 0.1% FA in DI water) ran for 3 minutes; the percentage of solvent B’ in solvent A’ ramped linearly from 1% to 28% over 70 minutes; the percentage of solvent B’ in solvent A’ ramped linearly from 28% to 45% over 25 minutes; the percentage of solvent B’ in solvent A’ ramped linearly from 45% to 90% over 12 minutes; 90% solvent B’ in solvent A’ ran for 5 minutes. MS/MS acquisition over the course of the method was performed in a data-dependent style (Top N=20, z=2-6, and threshold intensity =9100) with a dynamic precursor exclusion for 20 seconds after each scan. Both CID and HCD fragmentation spectra were acquired for each selected precursor ion. Orbitrap was used as a detection method for both primary (resolution=70000) and secondary (resolution=17500) mass spectra.

De novo peptide sequencing was conducted using PEAKS X+ from Bioinformatics Solutions Inc. Original mass spectrometry data were refined by merging HCD and CID scans within a 0.2-minute and 10 ppm window. Mass precursor correction and primary mass filtration were performed as needed. De novo sequencing enabled the corrections of assignment errors within 15 ppm and individual fragment mass errors within 0.05 Da. For the peptides of three-AAs composite letters, fixed post-translational modification used was amidation (C terminus, any residue, -0.9840 Da). Variable post-translational modifications used were Orn (Ala, anywhere, 43.0422 Da), Dab (Ala, anywhere, 29.0265 Da), Dap (Ala, anywhere, 15.0109 Da), Carbamidomethylation (Cys, anywhere, 57.0215 Da) and Oxidation (His, Trp and Met, anywhere, 15.9949 Da). For the peptides of six-AAs composite letters, fixed post-translational modifications used were alanine amidation (C terminal, any residue, 70.0531 Da) and acetylation (N terminus, any residue, 42.0106 Da). Variable post-translational modifications used were Orn (Ala, anywhere, 43.0422 Da), Dap (Ala, anywhere, 15.0109 Da), Carbamidomethylation (Cys, anywhere, 57.0215 Da) and Oxidation (His, Trp and Met, anywhere, 15.9949 Da). Five candidate sequences were obtained for each preprocessed scan.

**2.5. Calculation of coding density**

For composite mapping, the maximum coding density can be calculated by the following formula,

$p=\lfloor\log_{2} \left( C_{n}^{k} \right)\rfloor$,

where *n* is the total number of different AAs, *k* is the number of different AAs used for each composite letter, and ⌊ ⌋ means rounding down. Therefore, $2^{p}$ combinations are selected from $C_{n}^{k}$ combinations for mapping, and each k-AAs composite letter represents a p-bits binary code.

**2.6. Statistical analysis of sequencing results**

Original mass spectrometry data were processed by PEAKS X+ software. Original AAs in the peptide chains were substituted by corrected AAs according to Table S1 and the peptide chains were then screened by chain length and average local confidence (ALC). The probability of different AAs at each position was statistically calculated based on the peptide chains left. For k-AAs composite mapping, the composite letter at each position was consisted of k different AAs with the top k highest probability at the corresponding position.


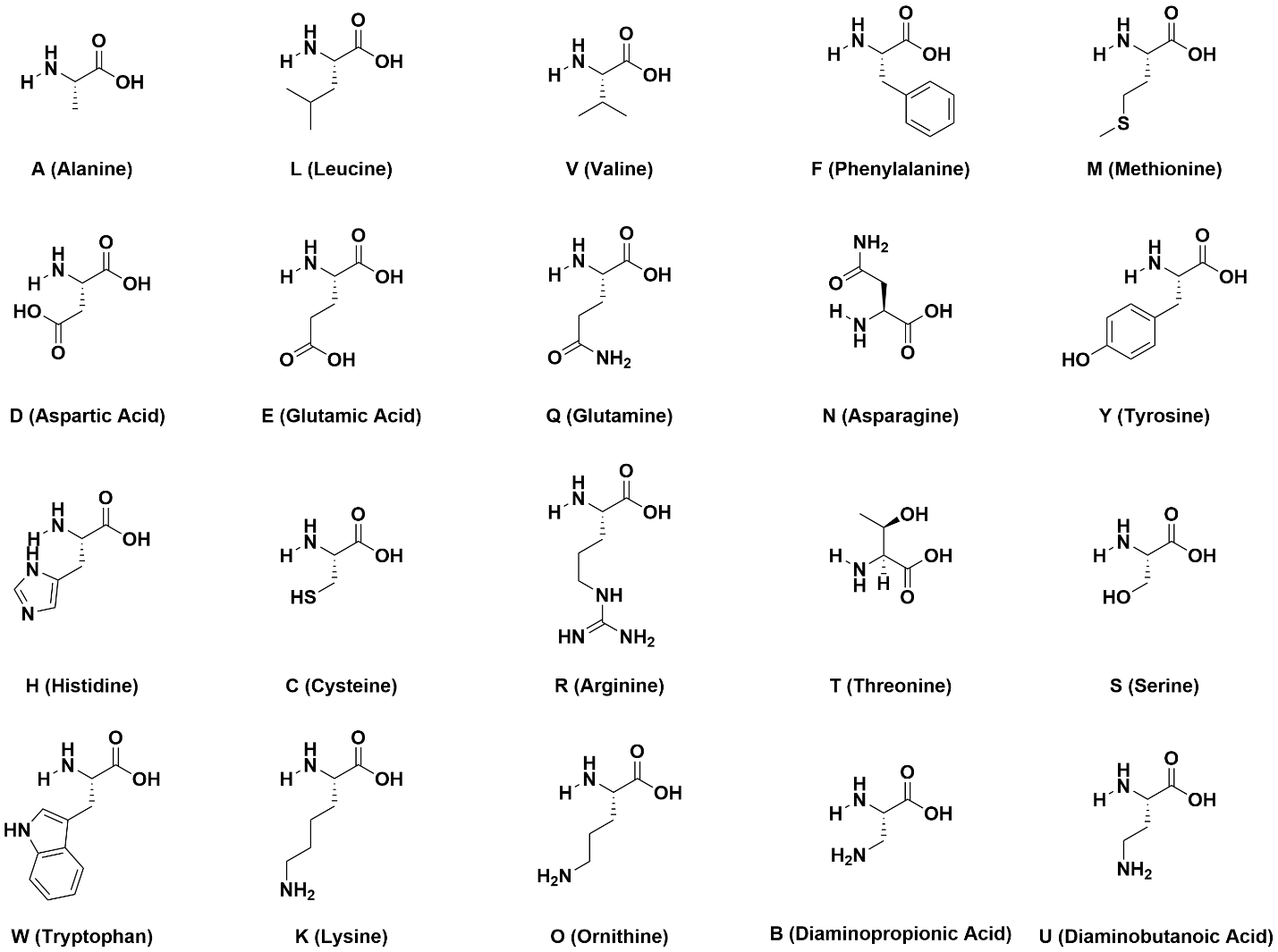


Figure S1. Structures and abbreviations of 20 amino acids (AAs) used for three-AAs composite letters. The factors considered in selecting the 20 AAs include mass, solubility, cost, synthesis and sequencing.


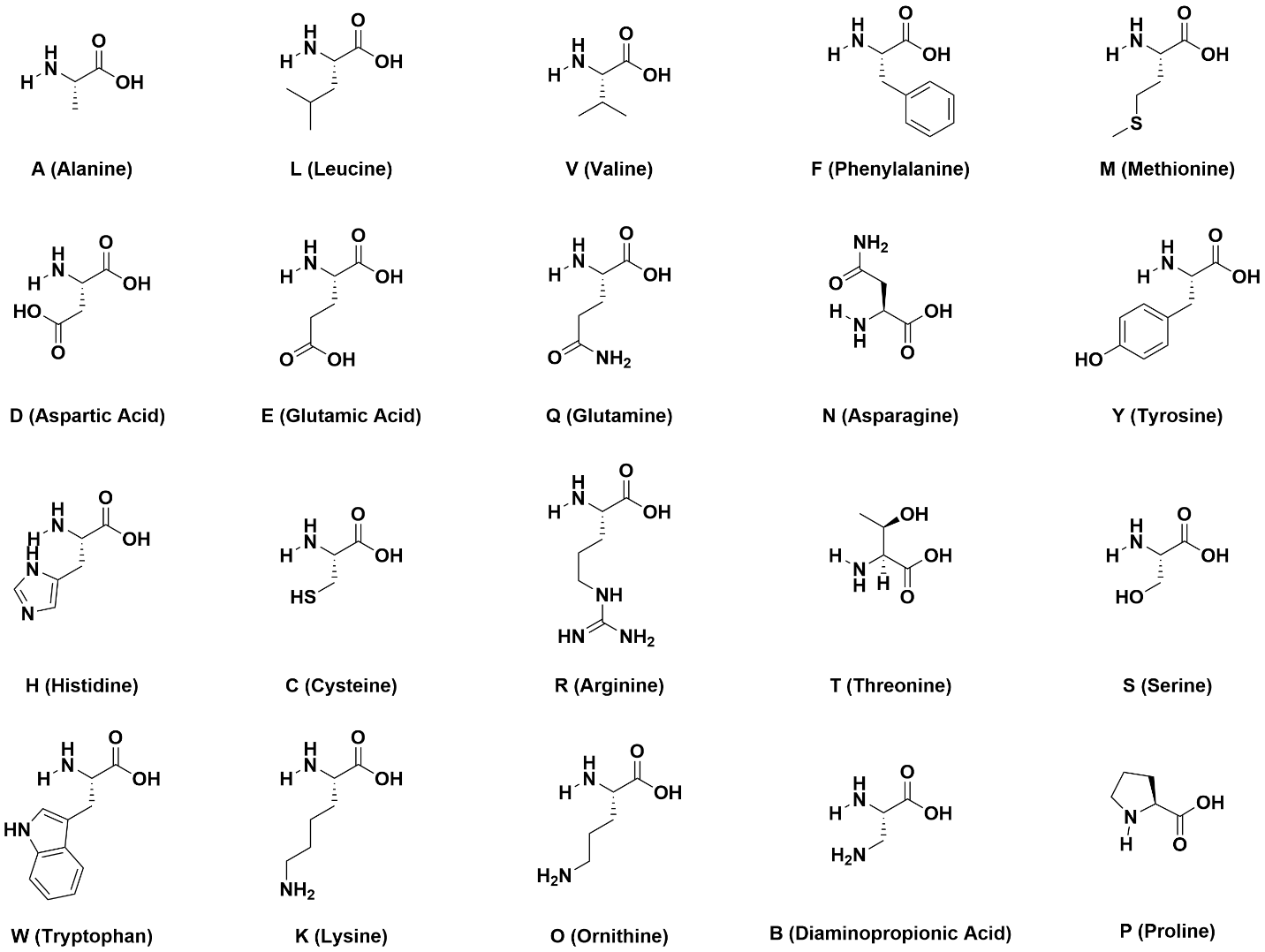


Figure S2. Structures and abbreviations of 20 AAs used for six-AAs composite letters. U used for three-AAs composite letters is replaced by P for six-AAs composite letters, as U is a non-canonical AA and exhibits poor sequencing accuracy.


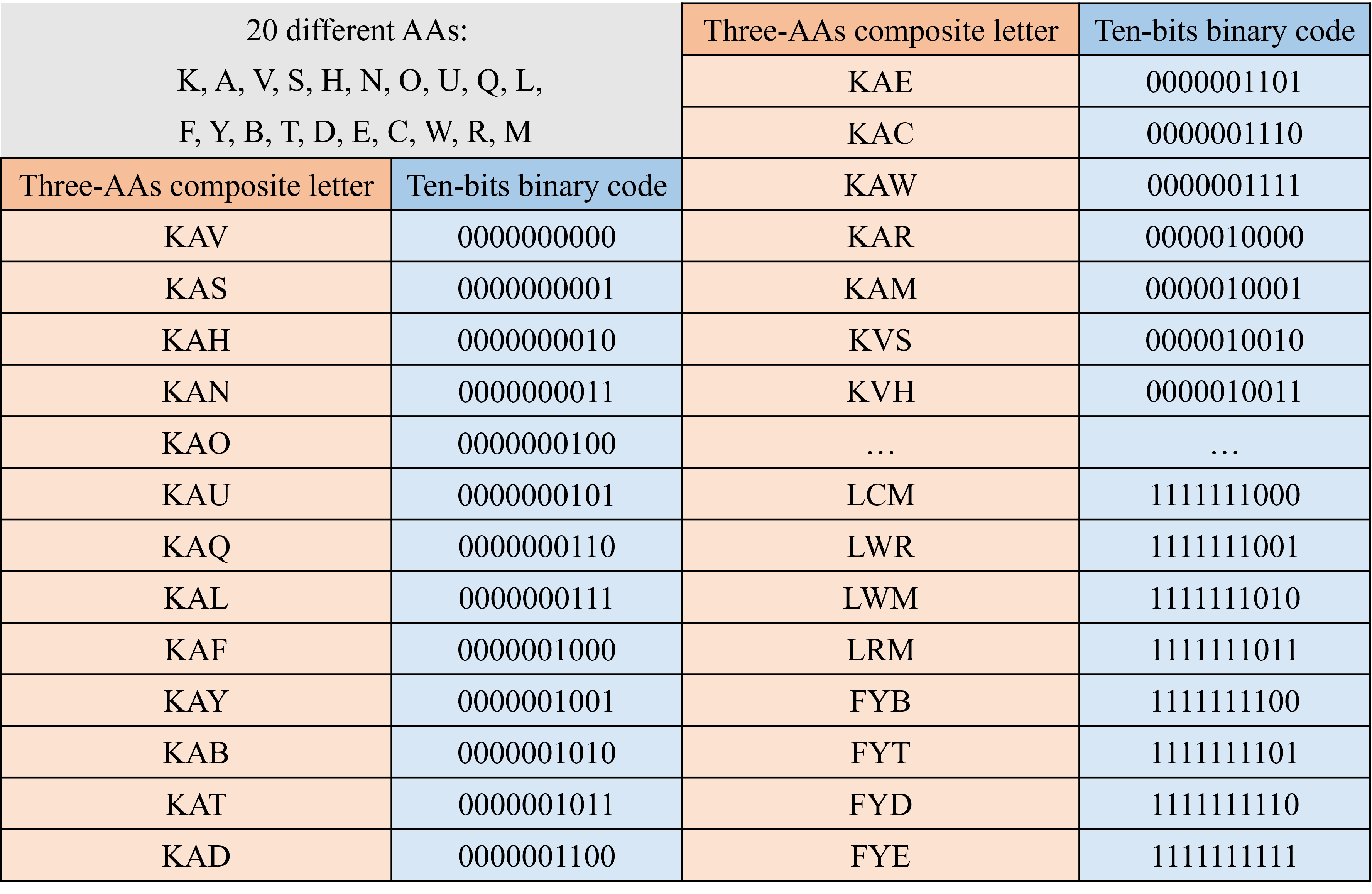


Figure S3. Mapping table of three-AAs composite letters to ten-bits binary codes. 2^10^ three-AAs composite letters selected from $\mathbf{C}_{\mathbf{20}}^{\mathbf{3}}$ combinations are mapped to 2^10^ ten-bits binary codes. If not specified, 20 different AAs are used for composite mapping. Three different AAs are selected consecutively from left to right of K, A, V, S, H, N, O, U, Q, L, F, Y, B, T, D, E, C, W, R and M. As the three-AAs composite letter changes from KAV to KAS, KAH…FYT, FYD and FYE, the ten-bits binary code increases from 0000000000 to 0000000001, 0000000010…1111111101, 1111111110 and 1111111111.


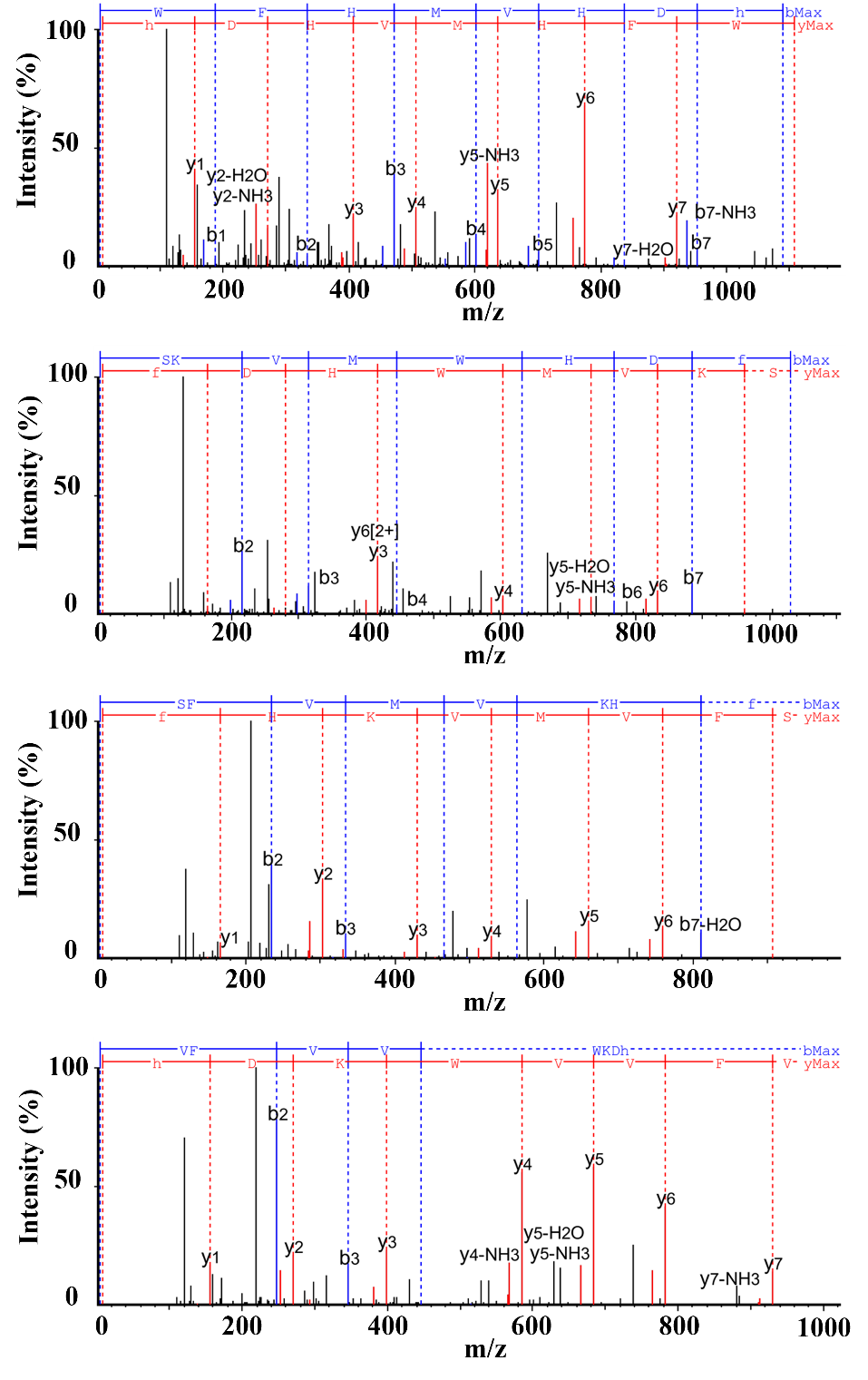


Figure S4. Mass spectra of 4 chains, i.e. “WFHMVHDH”, “SKVMWHDF”, “SFVMVKHF” and “VFVVWKDH”, from the sample of 8 three-AAs composite letters synthesized by mixed amino acids of equal molar mass.


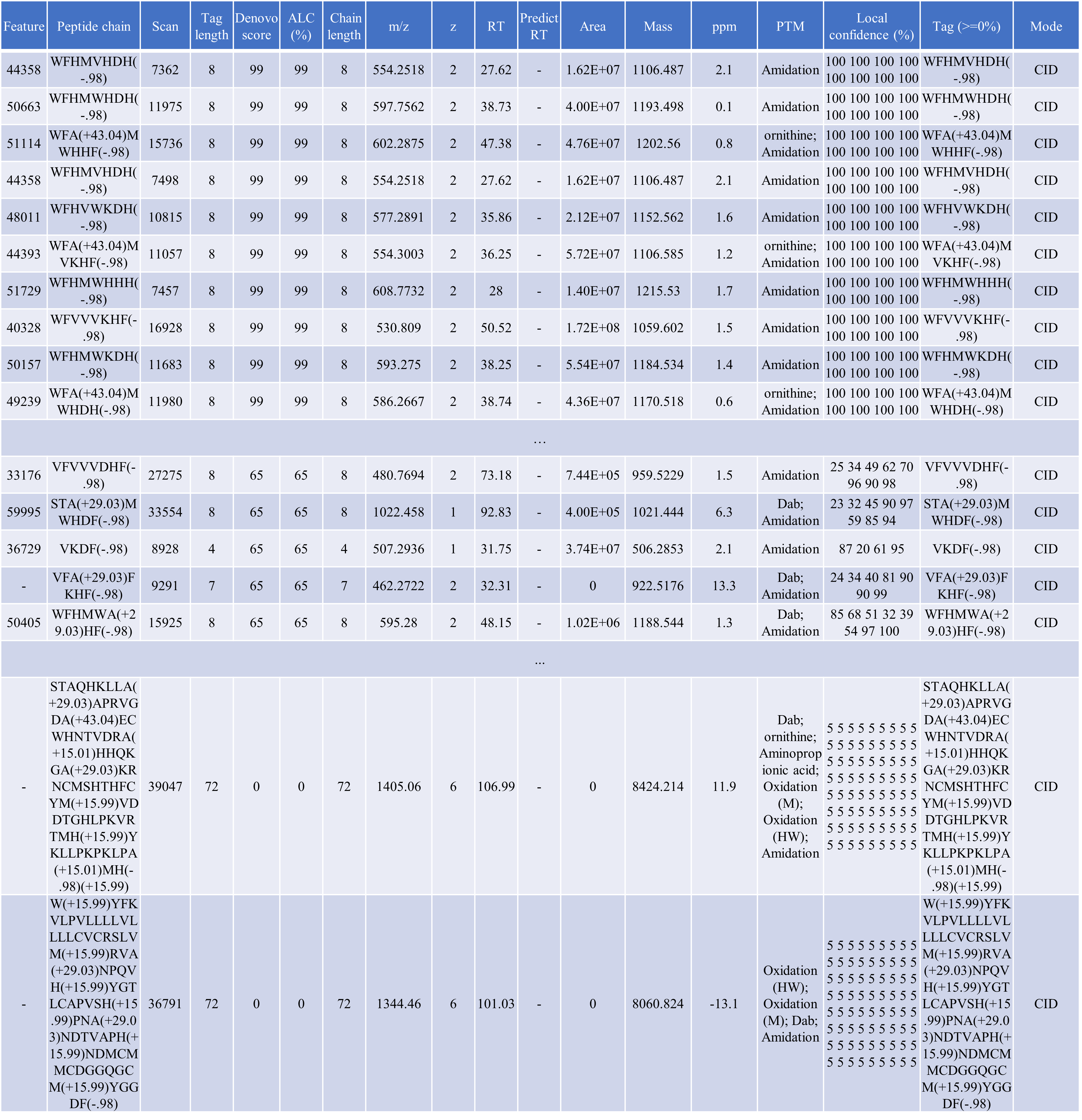


Figure S5. Peptide chains from the sample of 8 three-AAs composite letters synthesized by mixed amino acids of equal molar mass, which are obtained from mass spectrometry and analyzed by PEAKS X+ software.


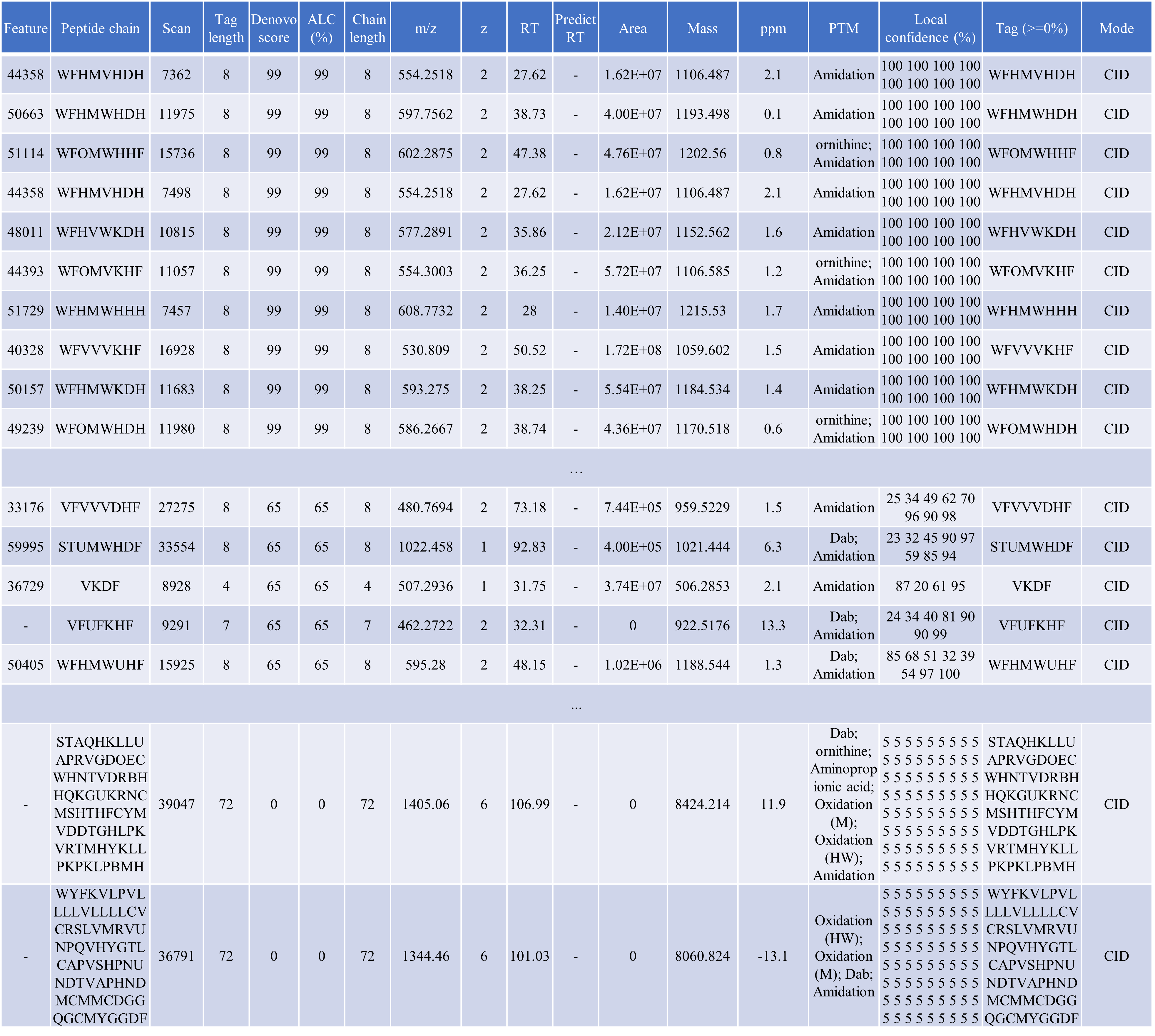


Figure S6. Peptide chains from the sample of 8 three-AAs composite letters synthesized by mixed amino acids of equal molar mass after substituting original AAs with corrected AAs according to Table S1.


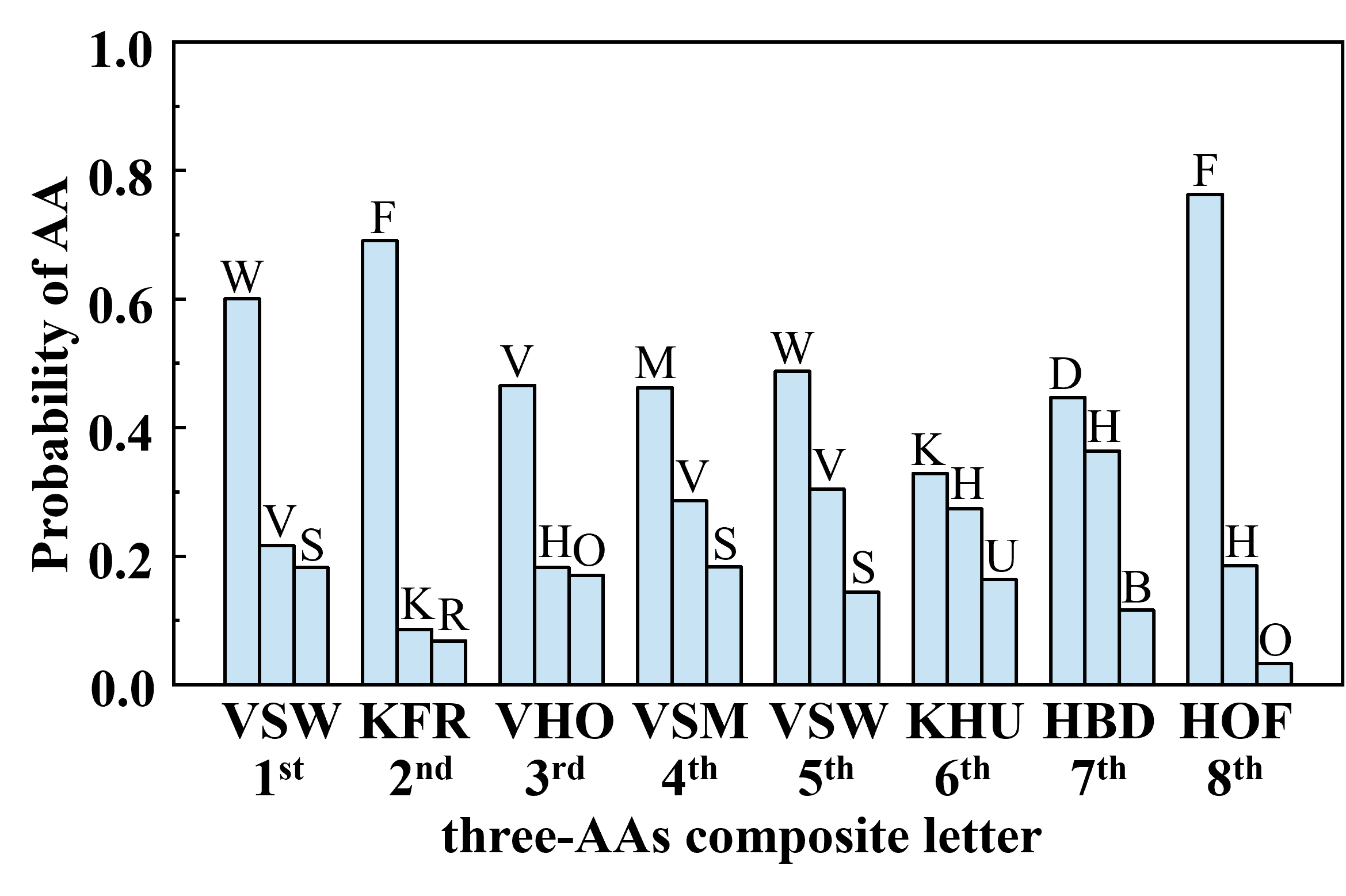


Figure S7. Probability of the three AAs with the top three highest probability at each position of the peptide sequence of 8 three-AAs composite letters synthesized by mixed amino acids of equal molar mass, when the average local confidence≥65%. The results indicate successful decoding.


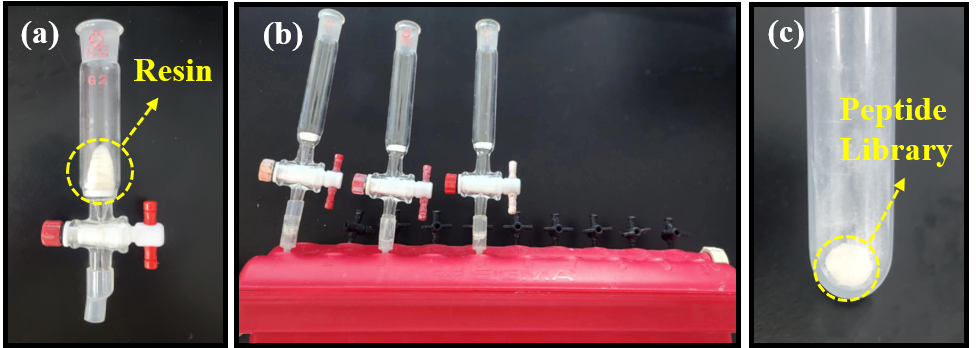


Figure S8. Photographs of (a) a 50 mL peptide synthesis tube containing resins, (b) three 10 mL peptide synthesis tubes and (c) a centrifuge tube containing synthesized peptides.


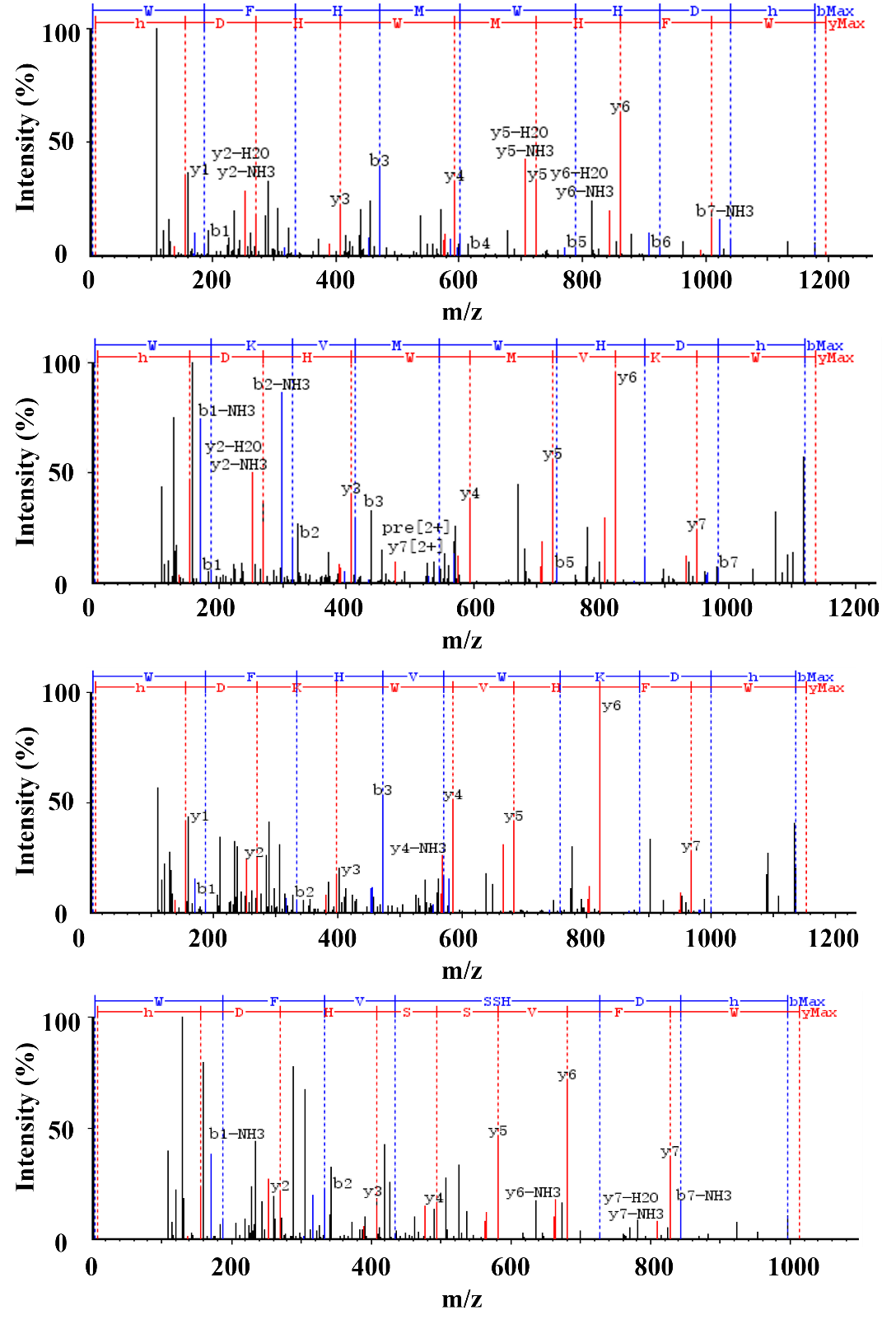


Figure S9. Mass spectra of 4 chains, i.e. “WFHMWHDH”, “WKVMWHDH”, “WFHVWKDH” and “WFVSSHDH”, from the sample of 8 three-AAs composite letters.


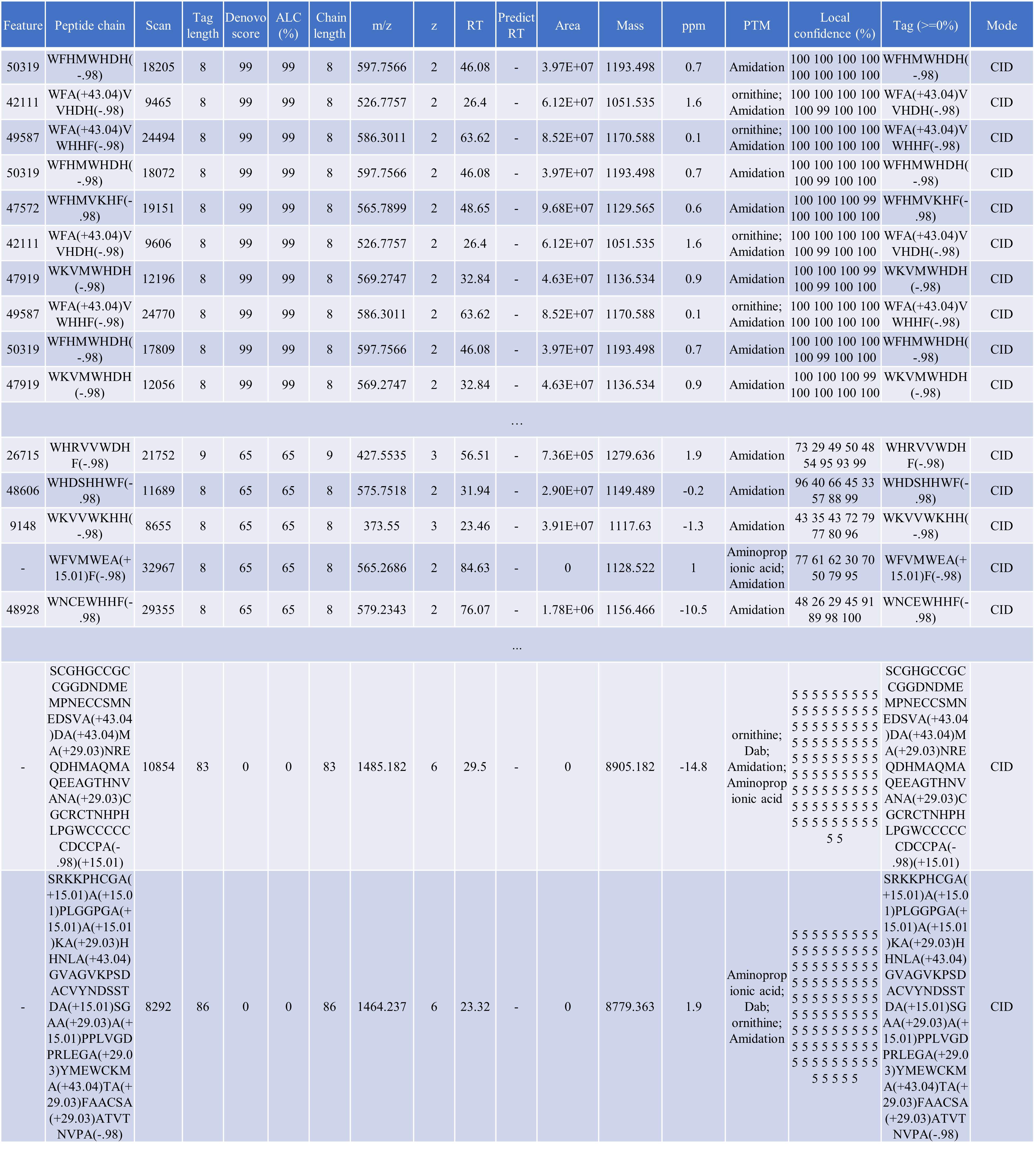


Figure S10. Peptide chains from the sample of 8 three-AAs composite letters obtained from mass spectrometry and analyzed by PEAKS X+ software.


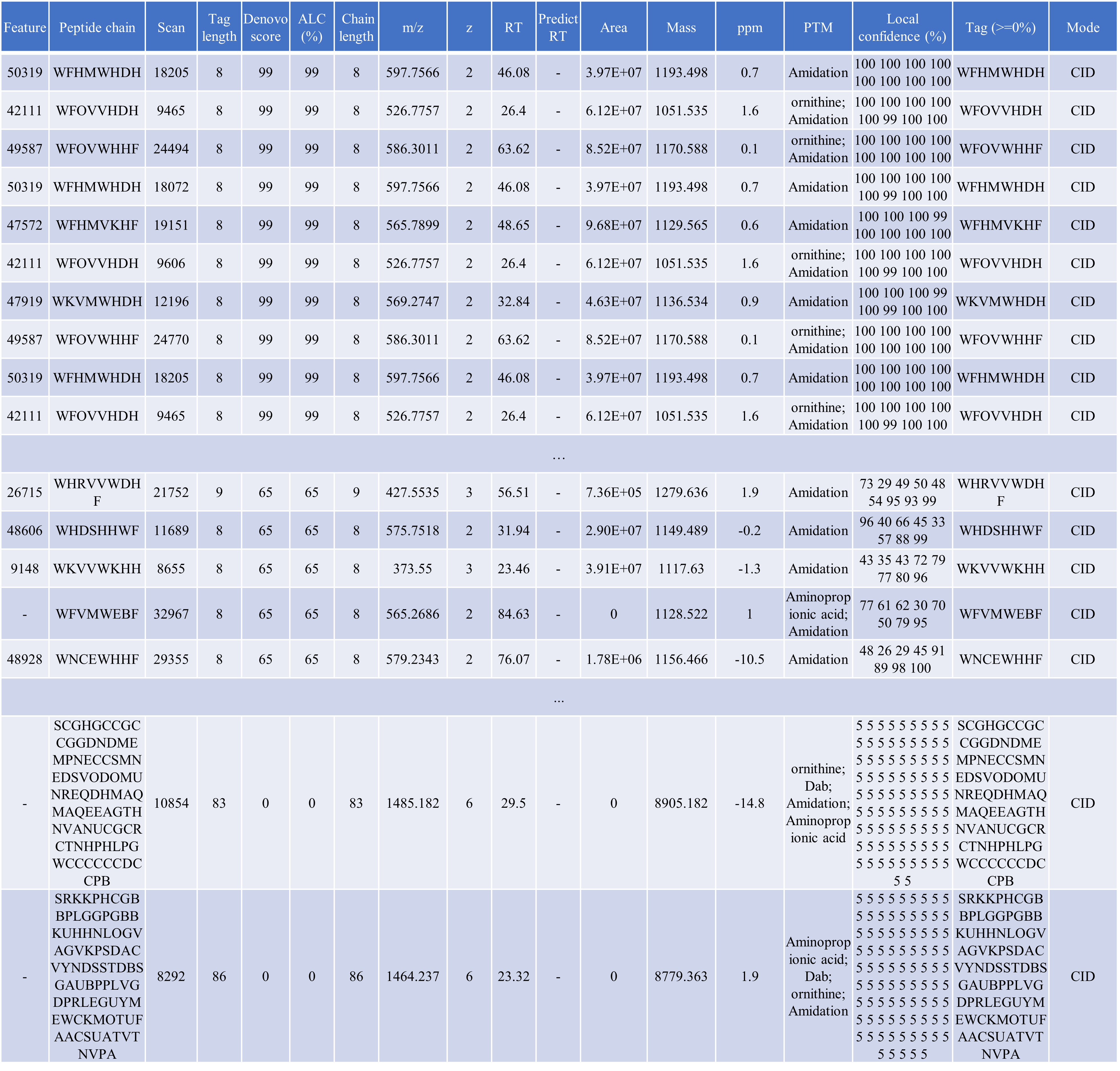


Figure S11. Peptide chains from the sample of 8 three-AAs composite letters after substituting original AAs with corrected AAs according to Table S1.


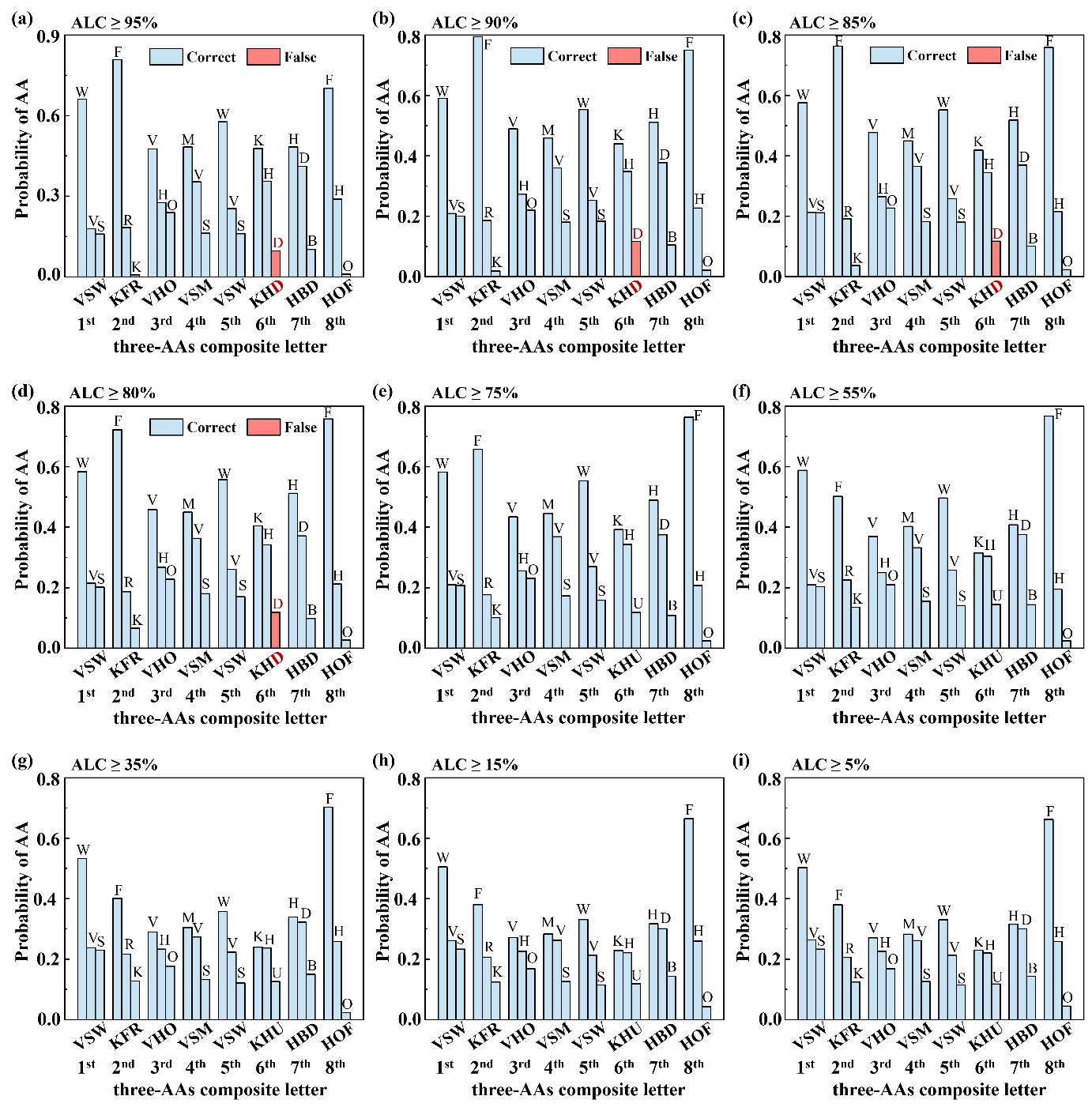


Figure S12. Probability of the three AAs with the top three highest probability at each position of the peptide sequence of 8 three-AAs composite letters, when the average local confidence (ALC) is (a) ALC≥95%, (b) ALC≥90%, (c) ALC≥85%, (d) ALC≥80%, (e) ALC≥75%, (f) ALC≥55%, (g) ALC≥35%, (h) ALC≥15% and (i) ALC≥5%. The three-AAs composite letter at each position is the combination of the three AAs with the top three highest probability at each position. A sequence of 8 three-AAs composite letters, i.e. VSW, KFR, VHO, VSM, VSW, KHU, HBD and HOF, is thus retrieved. The statistical results show that the 8 three-AAs composite letters retrieved when ALC≥75% or lower are all correct. Interestingly, the 8 three-AAs composite letters are all correct even when ALC≥5%, suggesting that the statistical results are robust against noises and errors.


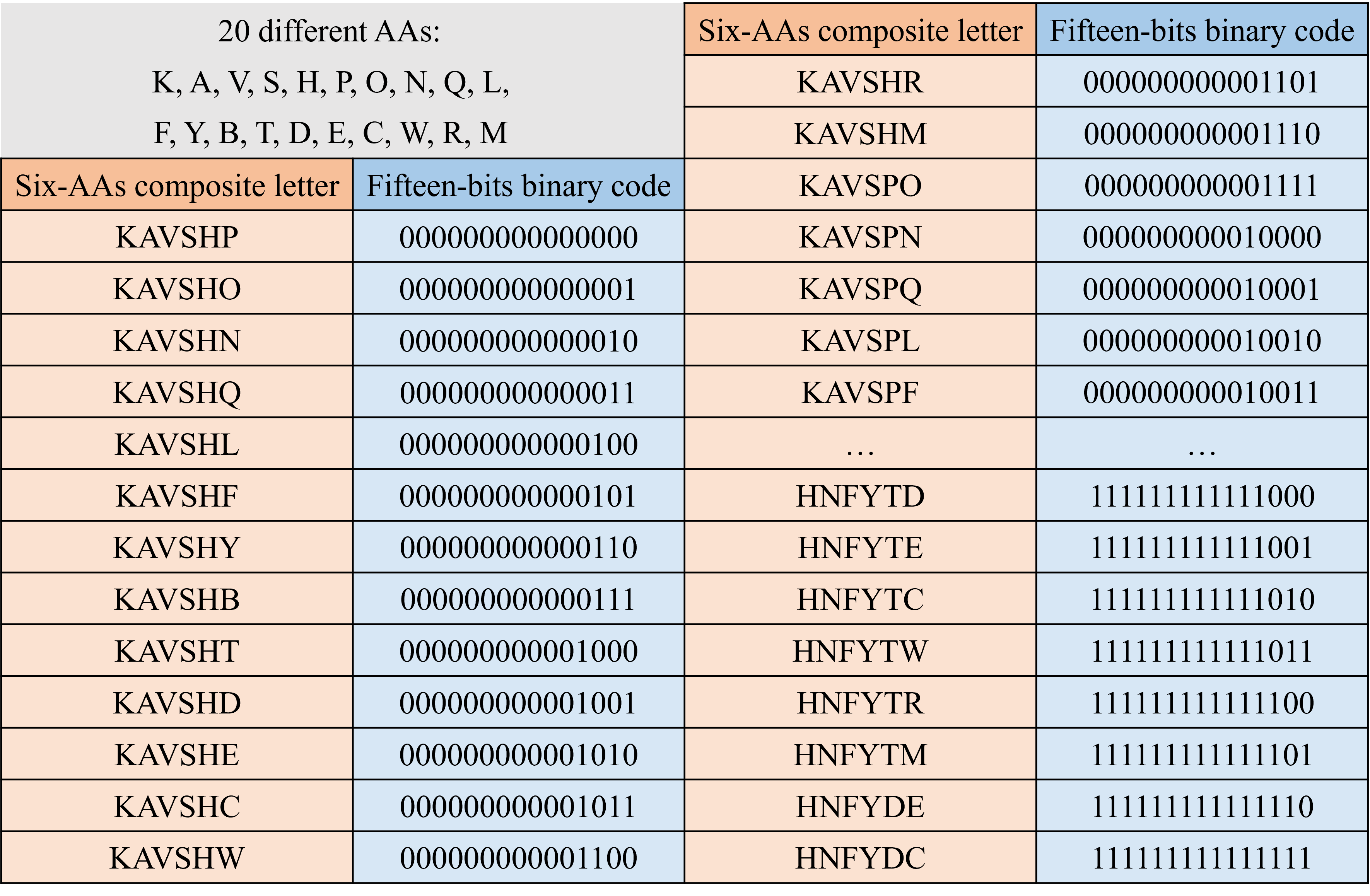


Figure S13. Mapping table of six-AAs composite letters to fifteen-bits binary codes. 2^15^ six-AAs composite letters selected from $\mathbf{C}_{\mathbf{20}}^{\mathbf{6}}$ combinations are mapped to 2^15^ fifteen-bits binary codes. Six different AAs are selected consecutively from left to right of K, A, V, S, H, P, O, N, Q, L, F, Y, B, T, D, E, C, W, R and M. As the six-AAs composite letter changes from KAVSHP to KAVSHO, KAVSHN…HNFYTM, HNFYDE and HNFYDC, the fifteen-bits binary code increases from 000000000000000 to 000000000000001, 000000000000010…111111111111101, 111111111111110 and 111111111111111.


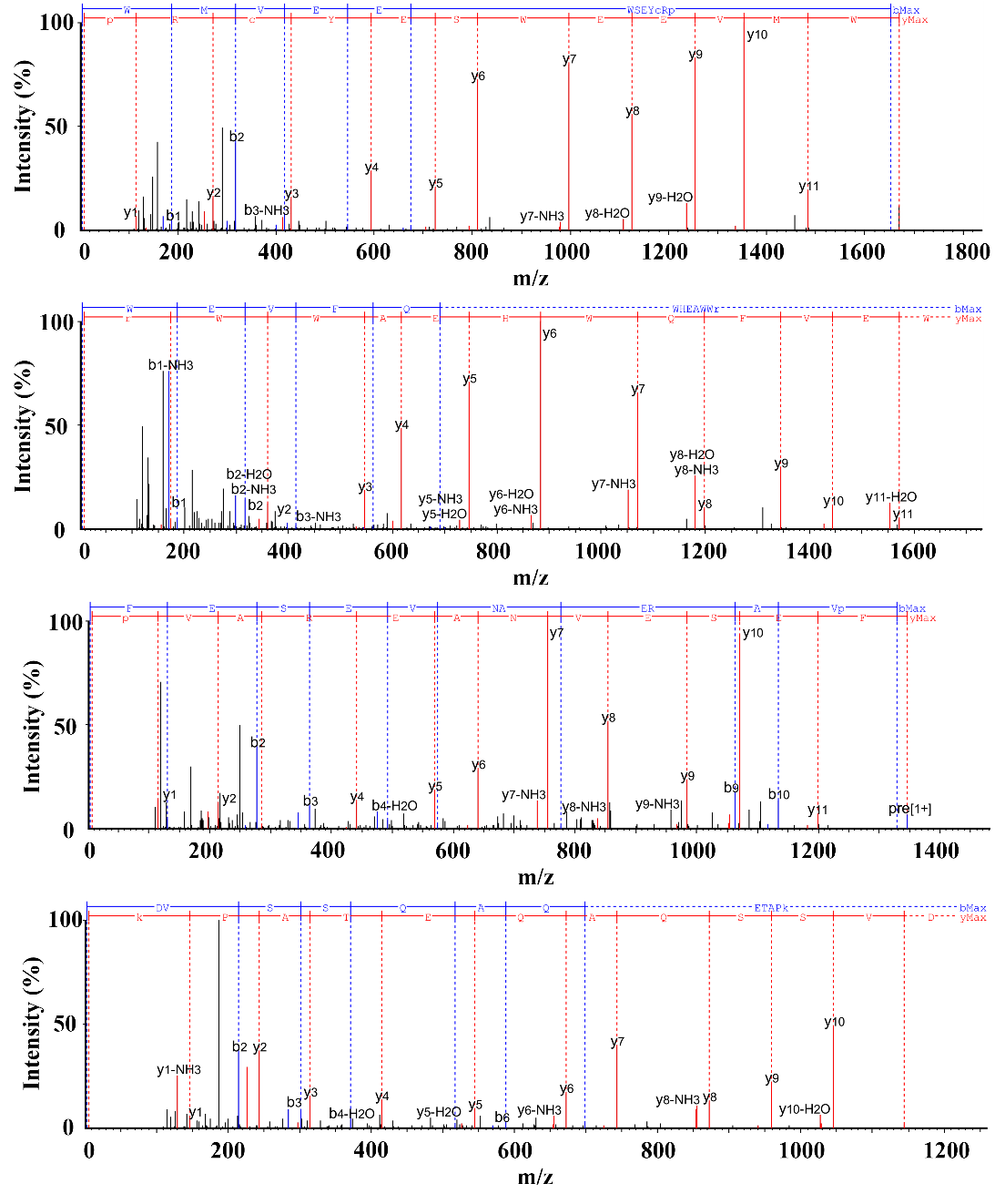


Figure S14. Mass spectra of 4 chains, i.e. “WMVEEWSEYCRP”, “WEVFQWHEAWWR”, “FESEVNAERAVP” and “DVSSQAQETAPK”, from the sample of 12 six-AAs composite letters before terminal modifications.


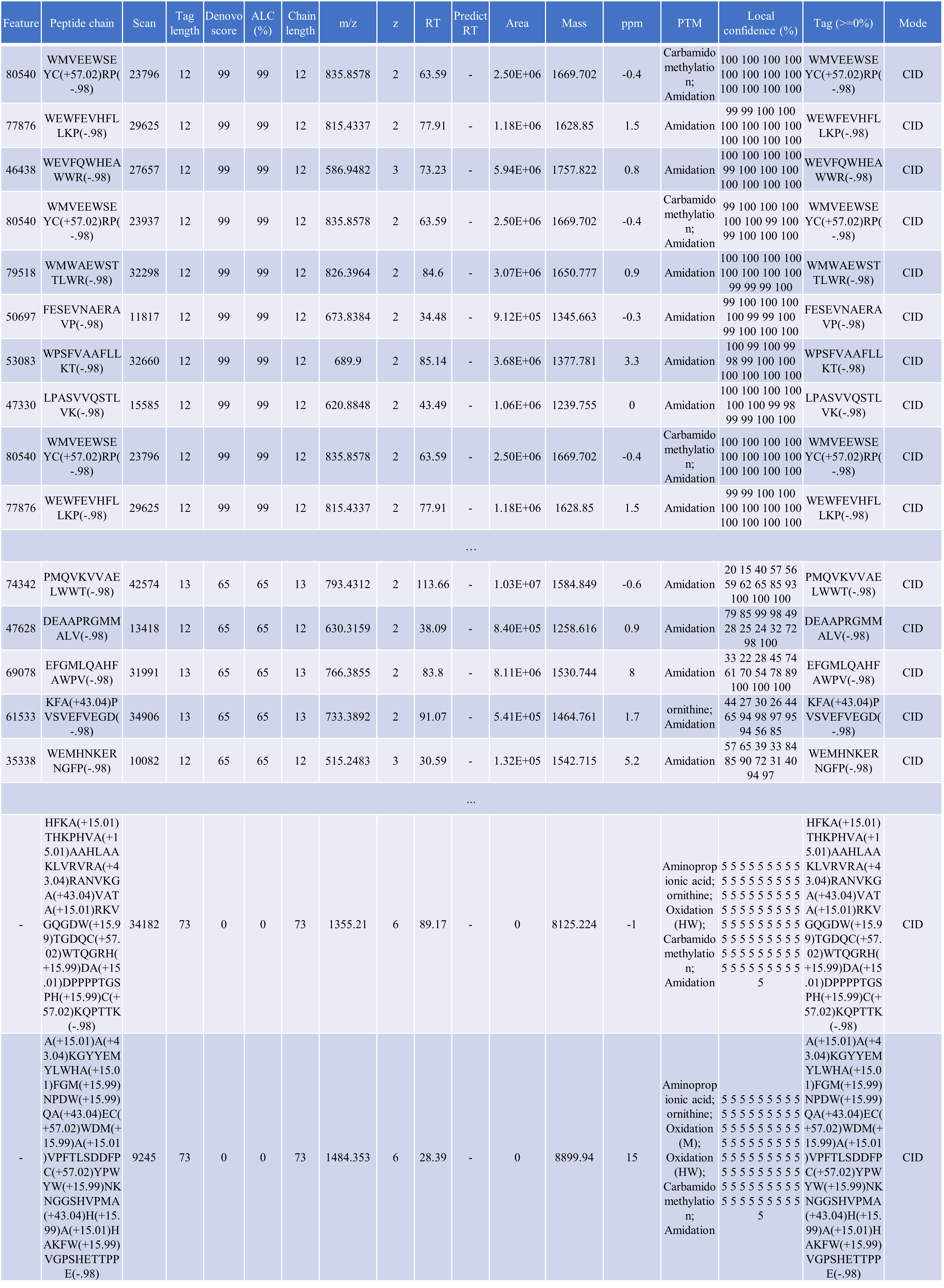


Figure S15. Peptide chains from the sample of 12 six-AAs composite letters before terminal modifications obtained from mass spectrometry and analyzed by PEAKS X+ software.


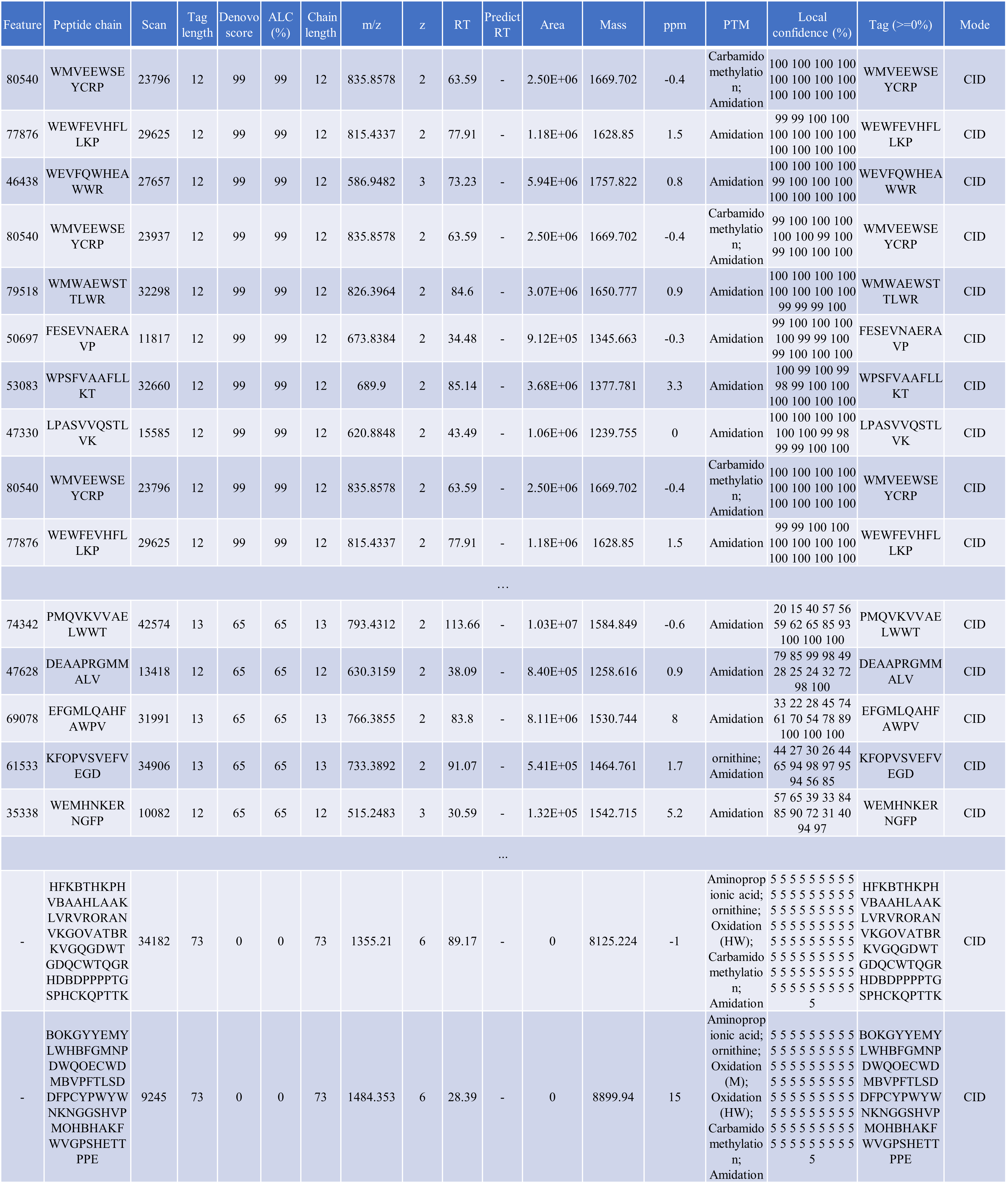


Figure S16. Peptide chains from the sample of 12 six-AAs composite letters before terminal modifications and after substituting original AAs with corrected AAs according to Table S1.


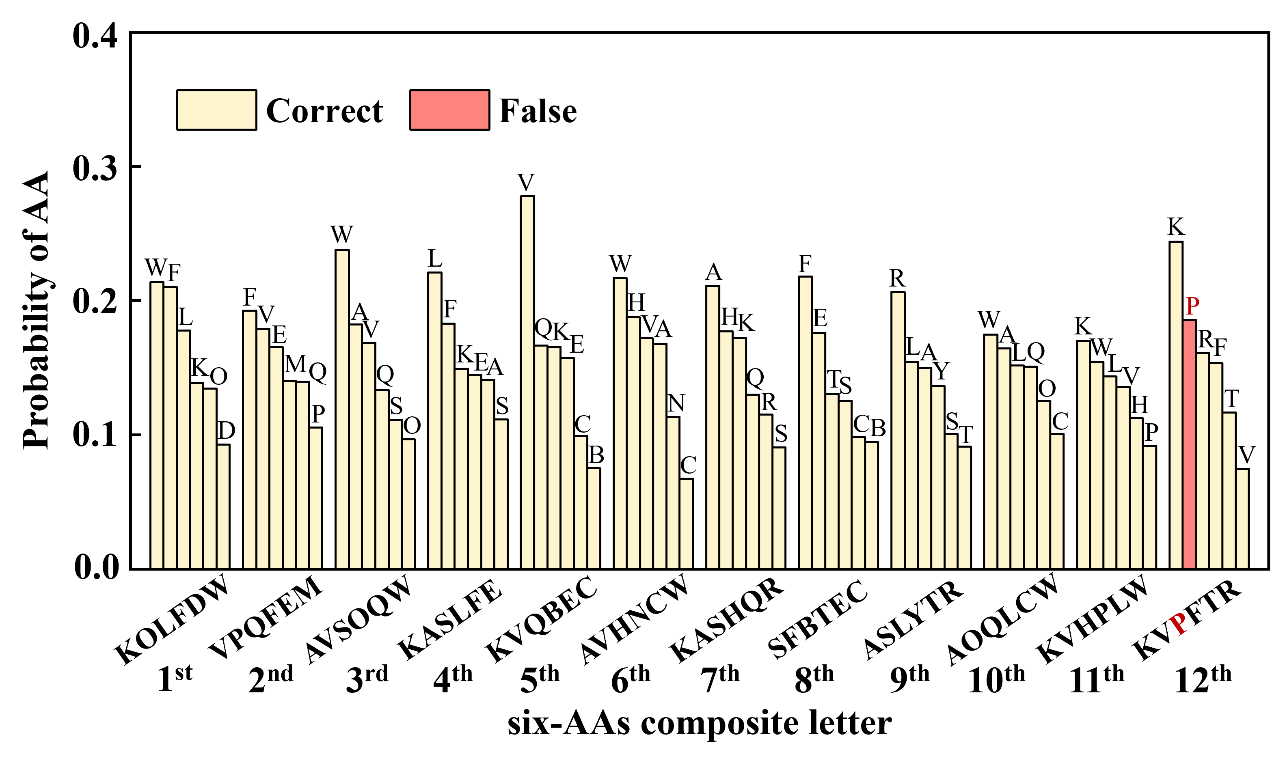


**Figure S17.** Probability of the six AAs with the top six highest probability at each position of the peptide sequence of 12 six-AAs composite letters after modifying the amino terminal. When the amino terminal is acetylated, only an error appears near the carboxyl terminal.


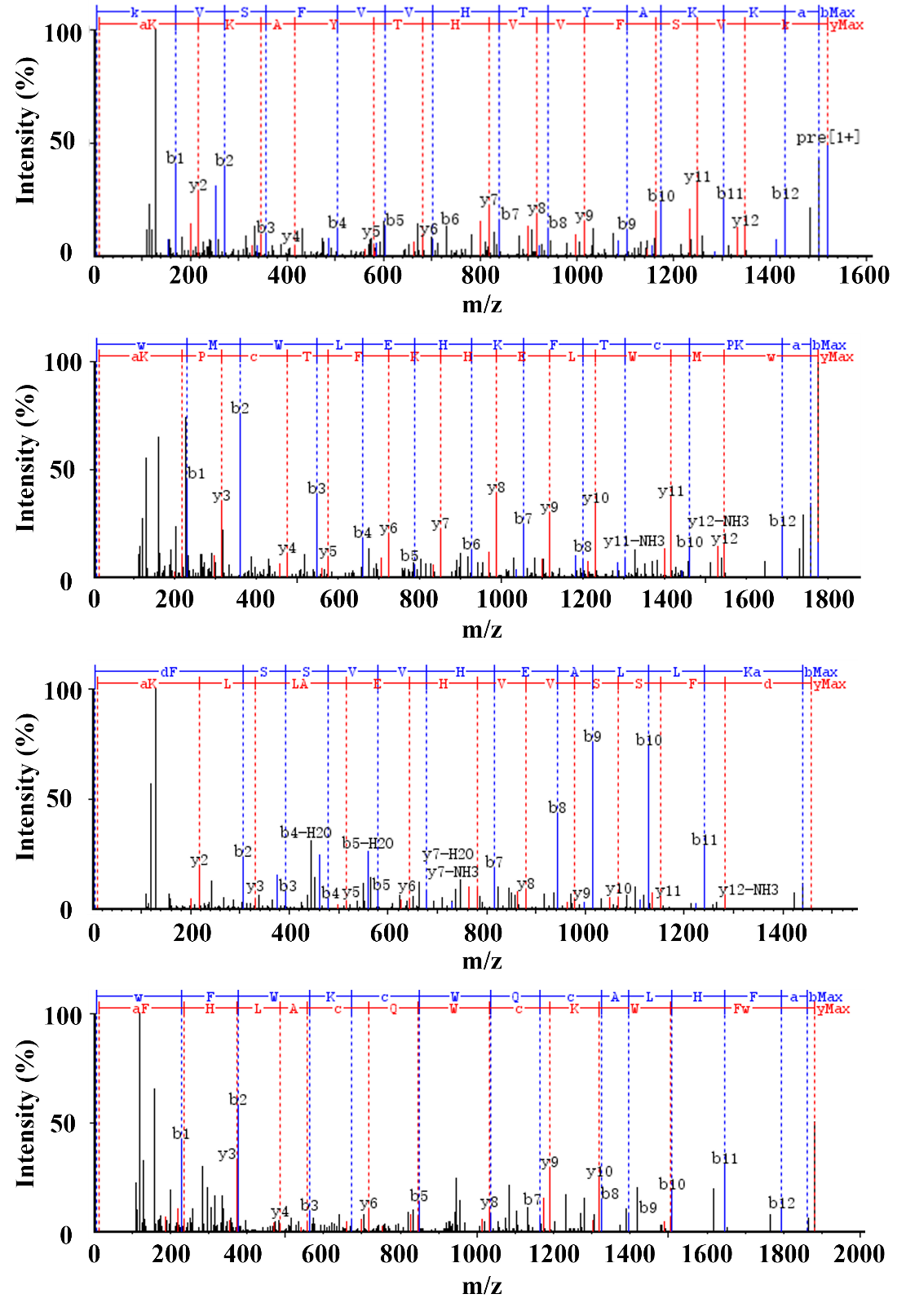


**Figure S18.** Mass spectra of 4 chains, i.e. “KVSFVVHTYAKK”, “WMWLEHKFTCPK”, “DFSSVVHEALLK” and “WFWKCWQCALHF”, from the sample of 12 six-AAs composite letters after terminal modifications.


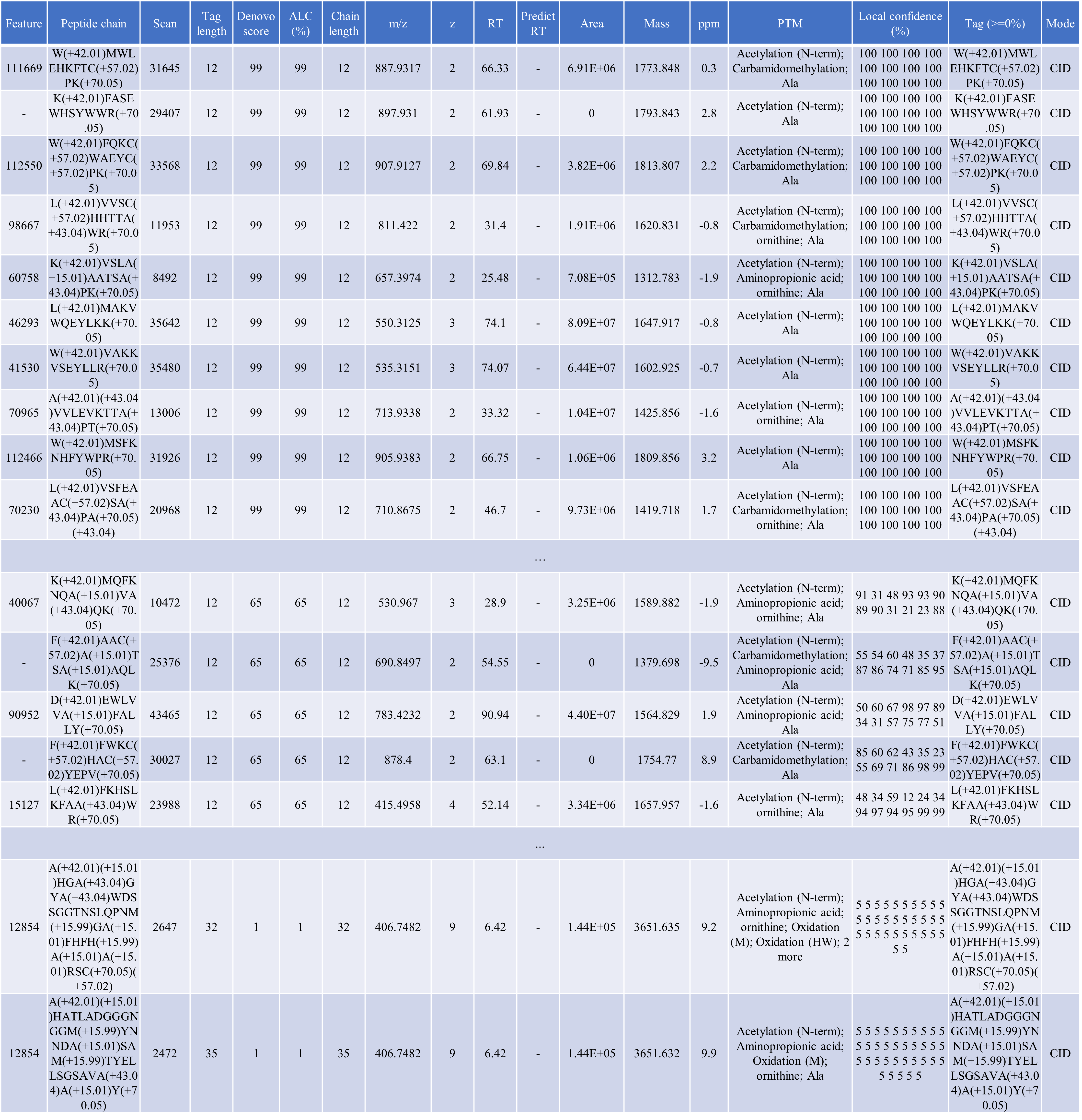


Figure S19. Peptide chains from the sample of 12 six-AAs composite letters after terminal modifications obtained from mass spectrometry and analyzed by PEAKS X+ software. +42.01 at the 1^st^ position is attributed to the modification with an acetyl group. +70.05 at the 12^th^ position is attributed to the modification with an alanine.


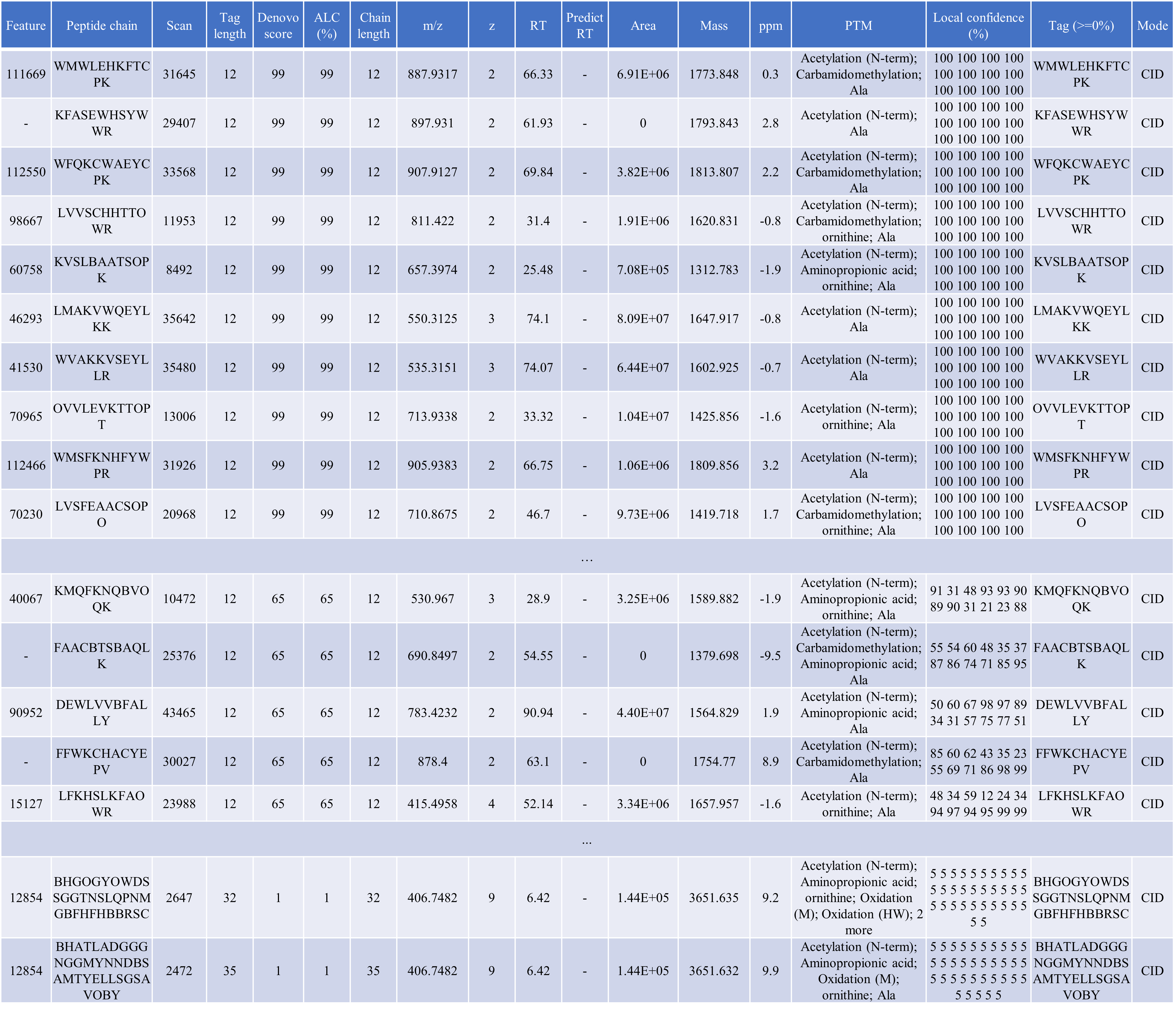


Figure S20. Peptide chains from the sample of 12 six-AAs composite letters after terminal modifications and after substituting original AAs with corrected AAs according to Table S1.


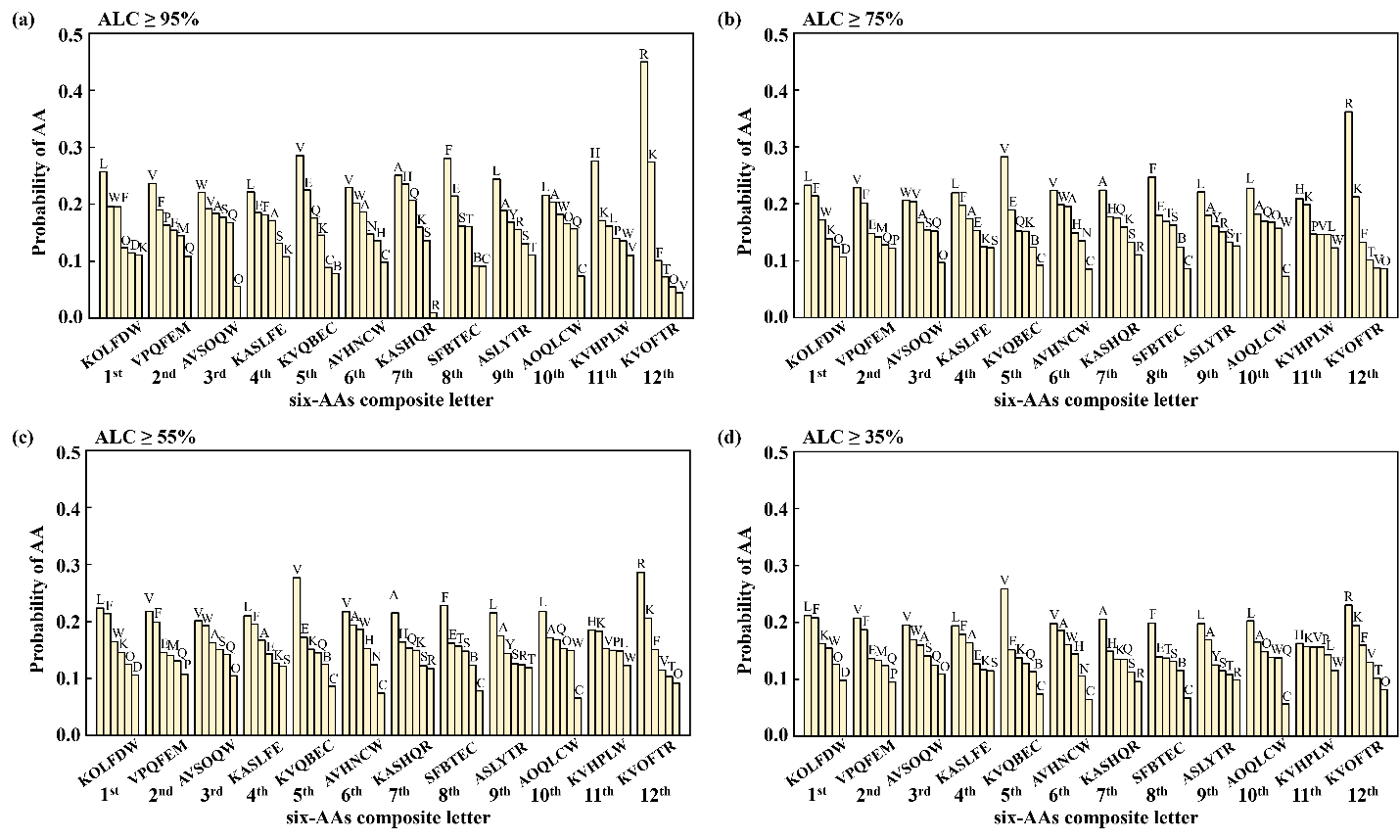


Figure S21. Probability of the six AAs with the top six highest probability at each position of the peptide sequence of 12 six-AAs composite letters, when (a) ALC≥95%, (b) ALC≥75%, (c) ALC≥55% and (d) ALC≥35%. The six-AAs composite letter at each position is the combination of the six AAs with the top six highest probability at each position. A sequence of 12 six-AAs composite letters, i.e. KOLFDW, VPQFEM, AVSOQW, KASLFE, KVQBEC, AVHNCW, KASHQR, SFBTEC, ASLYTR, AOQLCW, KVHPLW and KVOFTR, is thus retrieved.


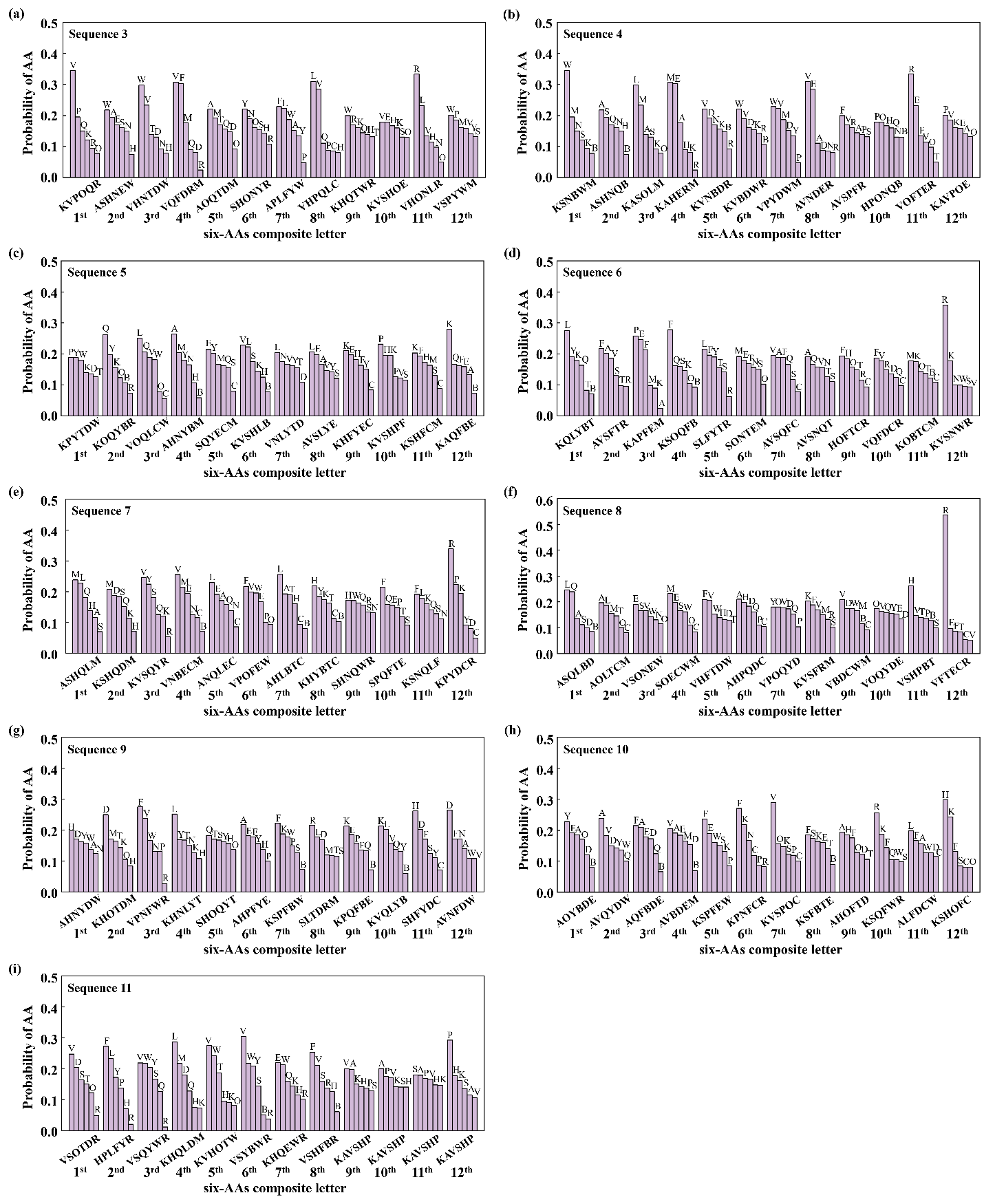


Figure S22. The 3^rd^, 4^th^, 5^th^, 6^th^, 7^th^, 8^th^, 9^th^, 10^th^ and 11^th^ sequences retrieved by statistical analysis. No errors are observed in all the 11 sequences. If not specified, AA chains are screened by chain length=12 and ALC≥65%.

Table S1. Substitution of original AAs with corrected AAs. Non-natural AA modifications and other protein translational modifications are considered to substitute original AAs with corrected AAs.

| Original amino acid | Corrected amino acid |
| --- | --- |
| A(+15.01) | B |
| A(+29.03) | U |
| A(+43.04) | O |
| X(+15.99) | X |
| X(+42.01) | X |
| X(+57.02) | X |
| X(-.98) | X |
| X(+70.05) | X |
| A(-.98)(43.04) | O |

X represents any AA.

Table S2. Comparison of the coding density of peptide-based data storage between previous works and this work.

|  | Ng et al.^[1]^ | | Zheng et al.^[2]^ | Ren et al.^[3]^ | | | This work | |
| --- | --- | --- | --- | --- | --- | --- | --- | --- |
| Tolerance to errors | Yes | | No | Yes | | | Yes | |
| Resistance to homopolymers | No | | No | Yes | | | Yes | |
| Other algorithms | Yes | | No | Yes | | | No | |
| Data type | Text | Audio | Text | Text | Figure | Audio | Text | Audio |
| Data size (bytes) | 106 | 1719 | 32 | 540 | 2673646 | 3897469 | 22 | 235 |
| Coding density (bits/AA) | 1.18 | 1.50 | 4.36 | 9.82 | 7.11 | 7.24 | 15.00 | 14.67 |

References

[1] C. C. A. Ng, W. M. Tam, H. Yin, Q. Wu, P. K. So, M. Y. Wong, F. C. M. Lau, Z. P. Yao, *Nat. Commun.* **2021**, 12, 4242.

[2] J. S. Zheng, J. Liang, W. W. Shi, Y. Li, H. G. Hu, C. L. Tian, L. Liu, *Sci. Bull.* **2021**, 66, 1542.

[3] Y. Ren, Y. Zhang, Y. Liu, Q. Wu, H. G. Hu, J. Li, C. Fan, D. Chen, K. Liu, H. Zhang, *Fundam. Res.* **2023**, 3, 298.
